# Evaluating the Environmental Sustainability of Alternative Ways to Produce Benzene, Toluene, and Xylene

**DOI:** 10.1021/acssuschemeng.3c06996

**Published:** 2024-03-18

**Authors:** Emma A. R. Zuiderveen, Carla Caldeira, Tijmen Vries, Niels J. Schenk, Mark A. J. Huijbregts, Serenella Sala, Steef. V. Hanssen, Rosalie van Zelm

**Affiliations:** † Department of Environmental Science, Radboud Institute for Biological & Environmental Sciences, Radboud University, Heyendaalseweg 135, 6525 AJ Nijmegen, The Netherlands; ‡ European Commission, Joint Research Centre, Via Enrico Fermi 2749, Ispra, 21027 Varese, Italy; § BioBTX B.V., Zernikelaan 17, 9747 AA Groningen, The Netherlands; ∥ Department of Circularity & Sustainability Impacts, TNO, Princetonlaan 6, 3584CB Utrecht, The Netherlands; ⊥ Syensqo Lyon Research and Innovation Center, 85 Avenue des Freres Perret, 69190 Saint-Fons, France

**Keywords:** aromatics, biobased chemicals, chemical recycling, prospective life cycle assessment, absolute sustainability

## Abstract

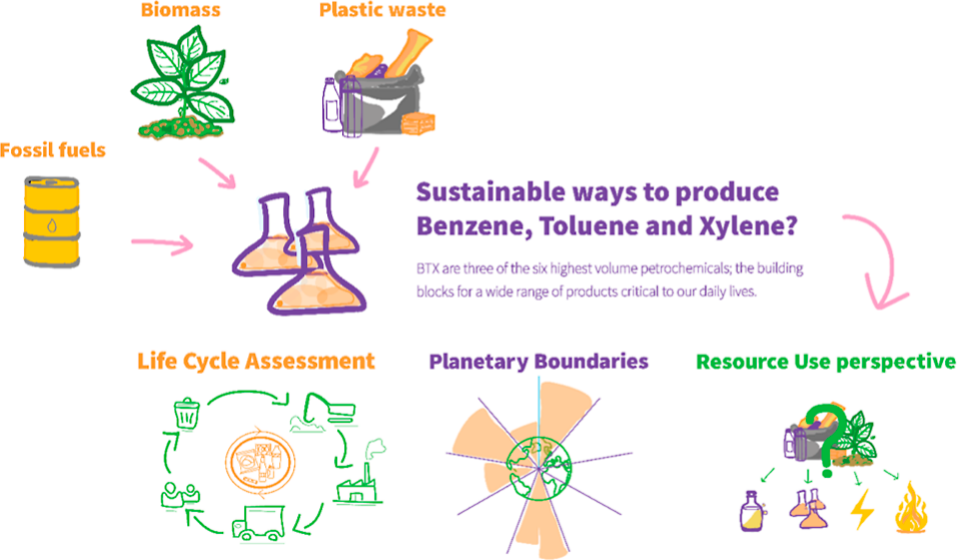

The petrochemical industry can reduce its environmental impacts by moving from fossil resources to alternative carbon feedstocks. Biomass and plastic waste-based production pathways have recently been developed for benzene, toluene, and xylene (BTX). This study evaluates the environmental impacts of these novel BTX pathways at a commercial and future (2050) scale, combining traditional life cycle assessment with absolute environmental sustainability assessment using the planetary boundary concept. We show that plastic waste-based BTX has lower environmental impacts than fossil BTX, including a 12% decrease in greenhouse gas (GHG) emissions. Biomass-based BTX shows greater GHG emission reductions (42%), but it causes increased freshwater consumption and eutrophication. Toward 2050, GHG emission reductions become 75 and 107% for plastic waste and biobased production, respectively, compared to current fossil-BTX production. When comparing alternative uses of plastic waste, BTX production has larger climate benefits than waste incineration with energy recovery with a GHG benefit of 1.1 kg CO_2_-equiv/kg plastic waste. For biomass (glycerol)-based BTX production, other uses of glycerol are favorable over BTX production. While alternative BTX production pathways can decrease environmental impacts, they still transgress multiple planetary boundaries. Further impact reduction efforts are thus required, such as using other types of (waste) biomass, increasing carbon recycling, and abatement of end-of-life emissions.

## Abstract 2

This study evaluates the environmental impact of alternative production of the petrochemicals benzene, toluene, and xylene combining different methods.

## Introduction

The petrochemical industry produces primary chemicals that form the building blocks for a wide range of products critical to our daily lives. At the same time, this industry is responsible for 7% of the global industrial greenhouse gas (GHG) emissions and accounts for 14% of the world’s oil demand.^1^ These impacts relate largely with the use of fossil fuels as carbon feedstock,^2^ consuming more than half of the sector’s fossil input.^3^ Therefore, shifting from fossil fuels to other carbon feedstocks, which includes biomass or recycled carbon sources, may reduce the GHG emissions and wider environmental impacts of this industry.^4^ At the European Union level, this shift has been advocated by several initiatives within the European Green Deal,^5^ including the chemical strategy^6^ and its link with climate ambition, circularity ambition, and overall sustainability of chemicals and materials.

Recently, novel production routes have emerged that use other carbon feedstocks for the aromatic petrochemicals benzene, toluene and xylene.^7^ These chemicals are known as BTX and account for 30 wt % of the current petrochemical production.^1^ One of these routes is catalytic pyrolysis, a process that utilizes heat to convert feedstock into oil and aromatics in the absence of oxygen.^8^ Feedstocks that can be used to produce BTX via catalytic pyrolysis are biomass-based,^9−11^ such as woody biomass or sugar cane bagasse,^11^ or plastic waste-based, such as high-density polyethylene waste.^12^

The few life cycle assessments (LCAs) on the environmental impacts of BTX production from alternative carbon feedstocks that have been performed mainly focused on climate change and have resulted in diverging outcomes. For biomass-based BTX, various authors have found lower GHG emissions for BTX from pulpwood compared to their fossil products,^10,13^ while BTX from wood chips in combination with CO_2_ capture may even result in negative emissions.^14^ In contrast, Lin et al.^15^ found higher GHG emissions for starch-based *p*-xylene compared to petroleum-based *p*-xylene. LCA studies that compare alternative treatments of mixed plastic waste showed that chemical recycling, i.e., using plastic waste to produce chemicals, results in lower GHG emissions than incineration with energy recovery.^16,17^ This finding points in the direction that BTX production might be a relatively climate-beneficial use of mixed plastic waste.^18^ How the plastic waste-based BTX is compared to fossil BTX is, however, still unknown.

A thorough understanding of the wider environmental impacts of BTX production from alternative carbon feedstock and how these routes are compared is currently lacking. In the European Union, the chemical strategy for sustainability has promoted a framework for safety and sustainability by designing chemicals and materials,^6,19^ recommending to address sustainability by means of LCA, and evaluating environmental impacts applying absolute sustainability concepts.^20^ An absolute sustainability assessment can determine if the alternative production routes are sustainable without transgressing the planetary boundaries. The planetary boundaries framework has approximated safe operating spaces for humanity with respect to the functioning of the Earth.^21^

The goal of our study is to comprehensively assess the environmental impacts of BTX production from biomass and mixed plastic waste at a projected commercial scale for the current situation (year 2024) and at a future industrial scale (year 2050). We contrast these pathways to BTX production from fossil fuels. A prospective LCA was carried out employing two impact assessment methods: the ReCiPe and the European Commission environmental footprint (EF). Additionally, the results were calculated adopting an absolute sustainability impact assessment method using the planetary boundary concept (PB-LCIA). We also explore the relative merits of using biomass and plastic waste as feedstocks for BTX production as compared to other common uses of these feedstocks.

## Materials and Methods

### Goal and Scope

**Figure 1:**
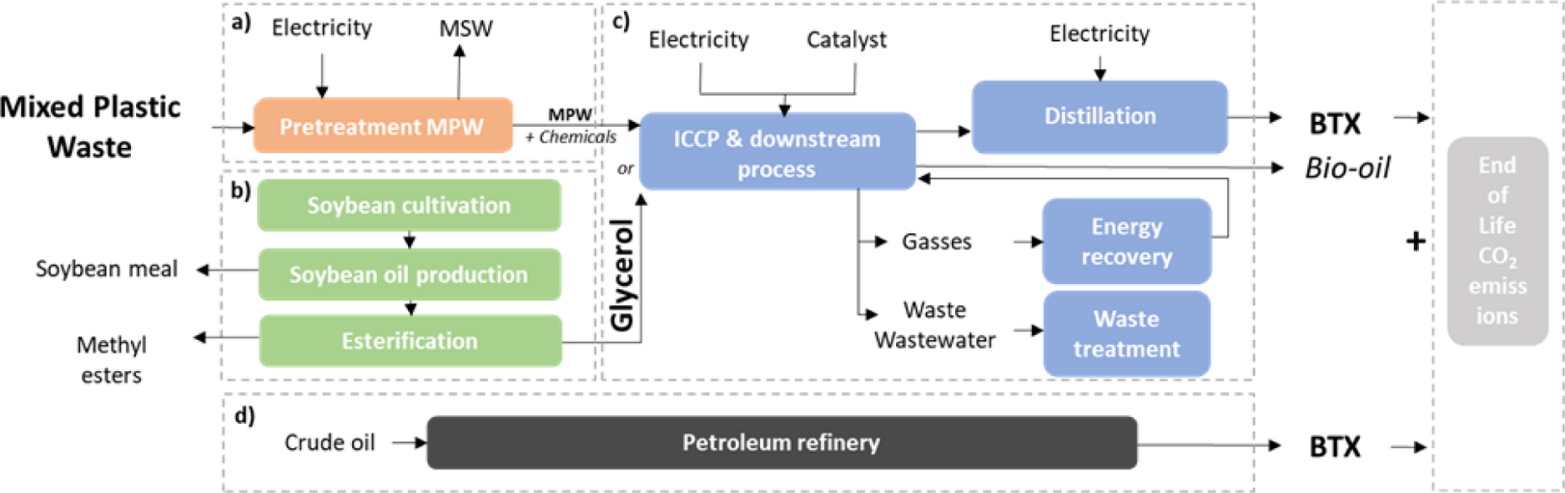
Simplified representation of benzene–toluene–xylene (BTX) production pathways including (a) mixed plastic waste (MPW) handling, i.e., pretreatment, (b) crude glycerol (biomass) production, (c) core processing for BTX production based on catalytic fast pyrolysis, and (d) petroleum refinery. ICCP = integrated cascading catalytic pyrolysis.

The goal of the LCA is to perform a comparative assessment to evaluate the environmental impacts of BTX production scaled at a commercial scale (TRL 9, 2024) and at a future industrial level scale (2050), using mixed plastic waste (DKR350), biomass (crude glycerol), and fossil-fuels (oil) as a feedstock. The base commercial scale scenario and future industrial scenario are further explained in section “Estimates of Future Life-Cycle Impacts” and Supporting Information S1.5. The BTX production pathways (Figure 1) from mixed plastic waste (MPW) and biomass are both based on the Integrated Cascading Catalytic Pyrolysis (ICCP) process developed by BioBTX B.V. (hereafter as BioBTX), a company located in Groningen, the Netherlands. In this process, the feedstock is first heated as the first step: the biomass and plastic molecules are cracked by heat, in the absence of oxygen. In the second step, the pyrolysis vapors released during this process are catalytically converted into aromatics, which are then separated from the noncondensable gases, and collected.^22^ For fossil BTX, the current conventional petroleum refinery route is included.^23^ The geographical scope is Europe for both the alternative BTX pathways and fossil-BTX, with the exception of specific processes that are known to occur in another part of the world (see Table 1).

**Table 1:** Life Cycle Inventory (LCI) Modelling Assumptions and Data Sources of MPW-BTX, Biobased BTX, and Fossil-BTX Pathways

inventory	modeling assumptions	source
mixed plastic waste	cut-off approach: no environmental impact allocated to its production only pretreatment	
biomass	glycerol as a byproduct of biodiesel production, economic allocation applied	Ecoinvent 3.8: glycerine {US}|esterification of soybean oil
transport (tkm)	MPW: default scenario of transport from sorting place to the plant of 50 km (0.05tkm)	Ecoinvent 3.8: transport, freight, lorry > 32 t, euro6 {RER}|market for transport, freight, lorry > 32 t, EURO6
	bio (glycerol): assumed transport from USA to the Netherlands (7.53tkm)	Ecoinvent 3.8: transport, freight, sea, container ship {GLO}|market for transport, freight, sea, container ship
pretreatment MPW	electricity for sorting of MPW: 0.250 MJ/kg MPW	Jeswani et al.,^29^ based on Krüger^31^
	electricity additional sorting of MPW: 0.058 MJ/kg MPW	Jeswani et al.^29^
catalyst	as zeolite-bentonite powder (20:80 weight ratio)	Ecoinvent 3.8: zeolite, powder (RER) production; activated bentonite (GLO), market group for
electricity (kW h)	used in different processes, in total 1 kW h/kg MPW and 1.05 kW h/kg glycerol	Ecoinvent 3.8: electricity, medium voltage {Western Europe}|market group for
distillation	based on energy needed for distillation (0.12–0.18 kW h/kg BTX) (Piccinno et al.;^34^ See Supporting Information S1.4)	Ecoinvent 3.8: electricity, medium voltage {Western Europe}|market group for
on-site gas system	energy recovery of waste gases, treated as natural gas. It covered 65 and 87% of electricity input for MPW- and biobased BTX, respectively. The on-site generated electricity required no additional fossil fuels. The carbon content of biobased BTX was considered biogenic	based on a combined heat and power (CHP), electricity from natural gas (Ecoinvent 3.8) and an electricity efficiency of 28%^35^
wastewater treatment	treating separated wastewater (0.26–1 kg/kg BTX)	Ecoinvent 3.8: wastewater, average {Europe without Switzerland}|treatment of wastewater, average
waste	MSW incineration (0.23–0.34 kg/kg BTX)	Ecoinvent 3.8: municipal solid waste {NL}|treatment of, incineration
fossil BTX	petroleum refinery (based a catalytic reformer and steam cracker)	Eco-profiles PlasticsEurope^23^
resource use perspective	incineration with energy recovery: MPW	Ecoinvent 3.8: waste plastic, mixture {CH}|treatment of, municipal incineration; lower heating value DKR-350 mix^28^ for energy recovery; Dutch incineration efficiencies^36^ (Supporting Information 1.8 for detailed data)
	biogas from glycerol	Stucki et al.,^37^ Ecoinvent 3.8: heat and power cogeneration, biogas {RER}. See Supporting Information 1.8 for detailed data
	purification glycerol	Cespi et al.,^38^ (Supporting Information 1.8 for detailed data)
	avoided products	Ecoinvent 3.8:
	bio-oil (0.08–0.14 kg CO_2_-equiv/kg BTX)	•light fuel oil {RER}|market for
	heat and electricity (0.5–1.1 kg CO_2_-equiv/kg MPW and 0.3–0.9 kg CO_2_-equiv/kg glycerol)	•heat, district or industrial, natural gas {RER}|market group for
	synthetic glycerol (3.1 kg CO_2_-equiv/kg glycerol)	•glycerine {RER}|production, from epichlorohydrin

The functional unit is “the production of 1 kg of mono-aromatics BTX”, and the system boundary was set to cradle-to-grave, including CO_2_ end-of-life emissions. BTX as a platform chemical has many applications.^24^ Therefore, we accounted for CO_2_ emissions by means of incineration, based on the chemical structure of BTX, but left all other waste treatment processes and emissions outside the system boundary. We included the CO_2_ end-of-life emissions to align with end-of-life biogenic and fossil carbon emissions, i.e., to include carbon uptake as well as its release. In the case of biobased BTX, the carbon content is considered neutral as it originates from short-rotating crops (soy),^25^ and for MPW and fossil BTX, the embedded carbon is fossil-based. The use phase was excluded from the assessment based on equivalence. Furthermore, to deal with the multifunctionality, economic allocation was applied as it reflects socio-economic demands.^26^ Allocation was performed for the byproducts soybean meal, methyl esters, and bio-oil, treated as light fuel oil, using 2011–2021 prices. Details on the methods are described in Supporting Information S1.

### Inventory

The alternative BTX production (currently at pilot-scale) is scaled to a commercial level (TRL 9, 2024) and to a future industrial level (2050). Table 1 shows the compiling of the inventories described and an overview of the modeling assumptions. The prospective and future scenario are further described in section “Estimates of Future Life-Cycle Impacts”. To model background processes, the Ecoinvent database (v3.8),^27^ system model “cut-off”, was used.

The mixed plastic waste used for BTX production was defined as “DKR-350”, based on the set of quality standards called “Deutsche Kunststoff Recycling”, which in the Netherlands represents the postconsumer mix of plastics that remains after the easily reusable plastics have been taken out.^28^ Following the “cut-off” approach, when MPW enters the system, it was assumed to have no environmental burden because it is a waste stream. This approach is often applied in studies on chemical recycling of plastic waste.^17,29,30^ Pretreatment impacts, i.e., sorting, were based on electricity needed to separate the plastics^31^ and an additional step to remove the impurities.^29^ Transport of MPW from the sorting facility to the plant was based on a EURO6 truck assuming an average 50 km transport distance.^31^

For the input of biobased BTX, crude glycerol production, the Ecoinvent process “glycerine esterification of soybean oil” from soybeans based on economic allocation from the USA was used. Following the PAS2050 guidelines,^32^ land use change emissions were assumed here to be zero for soybean production because it is on a land that has not changed land use over the past 20 years. Glycerol transport by containership from USA to Europe was included. Crude glycerol can then directly be fed into the reactor without further pretreatment.

The ICCP process was obtained from BioBTX. The processes of biomass and MPW into BTX differ in energy and chemical demand, but the catalyst use is similar. For the catalyst, zeolite powder in combination with bentonite,^33^ i.e., clay, was taken from Ecoinvent in a 20:80% ratio. Additionally, an on-site gas system was assumed to be installed for electricity generation to use the industrial plant’s byproducts. An additional distillation step was applied to the BTX output to produce the monoaromatics for further downstream uses. Here, the energy for a distillation step was calculated using the work of Piccinno et al.^34^ (Supporting Information S1.4). Impacts from process waste was treated as municipal solid waste and incinerated. Wastewater was assumed to be treated according to the Ecoinvent process “average wastewater treatment” in Europe.

The fossil BTX pathway was modeled based on the Eco-profiles of PlasticsEurope on petroleum refining, producing benzene, toluene and xylene in a 48:33:19 weight ratio. This ratio was assumed to be the same for biobased BTX and MPW-BTX.

### Prospective Analysis

To project the maturing of the alternative BTX pathways from pilot to a commercial and an industrial level, we followed the framework by van der Hulst et al.,^39^ which is a systematic procedure to assess future impacts of emerging technologies (Supporting Information S1.5). To go from pilot to a commercial level, the product output was scaled to 48 kton/year and process changes were introduced, including downstream steps, increased yield and energy input, and heat recovery (details can be found in Table S3 in Supporting Information S1.5). The industrial level (2050) included possible future external developments:

- Improvements due to technological advances were captured as improvements in energy intensity, assuming a reduction in the energy input of 1% per year.^40−42^
- Assessment of external developments for 2050 in the electricity sector was based on projections from the integrated assessment model IMAGE. IMAGE is an integrated assessment model to assess complex, large-scale environmental and sustainable development scenarios. Within this model, a future electricity mix is modeled based on drivers, such as costs and climate targets.^43^ Future developments were based on the shared socioeconomic pathway (SSP) 2 representing a middle-of-the-road narrative committed to a long-term climate target of 2.6 W/m^2^ in 2100 (SSP2 RCP2.6), consistent with the 2-degree target.^44^ The background data sets for the projected electricity market were systematically adapted using the approach of Mendoza Beltran et al.^45^
- While in the commercial (2024) scenario, we accounted for CO_2_ emissions by means of incineration of plastic waste at the end of life, this practice is likely to be reduced in the future.^46^ We followed the 2 °C-Circular Economy scenario on plastic flows based on the reports by Stegmann et al.^47^ for the future (2050) scenario, assuming only 13% of plastic waste is burned or used for energy and 87% of the embodied carbon remains in the loop.^47^ We assumed that all the end products BTX is used for are plastics.

### Life Cycle Impact Assessment (LCIA)

We applied two LCIA methods: ReCiPe2016 endpoint (H) and midpoint (H) (V1.1) and the environmental footprint (EF) method. For the absolute environmental sustainability assessment, we implemented the PB-LCIA method. These methods are further explained below.

### Mid- and Endpoint Assessment

To determine environmental impacts at both mid- and endpoint levels, the ReCiPe2016 endpoint (H) and ReCiPe midpoint (H) (V1.1)^48^ impact assessment methods were selected. A contribution analysis was done to research the contributions of the different processes and similarly to identify the contributions of the midpoint indicators to each endpoint indicator. The assessments were carried out in the Activity Browser,^49^ an open source LCA software built on BrightWay.^50^ At the midpoint level, we conducted an additional analysis using the EF method.^51^ This is the current method recommended by the European Commission for performing an LCA^32^ which is also included in the context of the environmental sustainability step of safety and sustainability by design recommendations.^20^

### Absolute Environmental Sustainability Assessment

To evaluate the environmental impacts in relation to the planetary boundaries,^52^ we applied the planetary boundaries life cycle impact assessment (PB-LCIA) method. This method introduces PB-informed characterization factors^53^ to connect to the elementary flows of the LCI and to map them onto the planetary boundaries’ safe operating spaces.^52,54^ Nine PBs are defined in total, but we excluded novel entities and atmospheric aerosol loading because they have not yet been adequately defined. For biosphere integrity, we followed the approach proposed in the reports by Galán-Martín et al. (2021) and updated it with more recent mean species abundance values from GLOBIO 3.5.^55,56^

The PB-LCIA results were compared with a safe operating space apportioned to the level of the product, i.e., 1 kg of BTX. For this downscaling, we applied a two-step method that first allocates the safe operating space to individuals and then to the product.^54,57^ We followed the approach described by Tulus et al.,^58^ defining a planetary boundary transgression level based on global population size and the price of BTX. Details on the PB-LCIA method are summarized in Supporting Information S1.7.

To compare the results of the PB-LCIA method, another PB-based approach was used as well: a normalization-based method that adapts the PB-framework to the impacts of the LCIA method. Here, we applied the carrying capacity-based normalization factors for the environmental footprint midpoint categories^59,60^ (Supporting Information Table S11).

### Sensitivity Analysis

To test the robustness of the results, sensitivity analysis on key parameters and modeling choices were carried out. In general terms, the allocation strategy is crucial. In terms of material requirements, the glycerol source in biobased BTX is especially relevant, while the plastic waste input in MPW-BTX has no impact. In terms of production, electricity is key as well as yield, which represents both efficiency and energy requirements. In terms of EoL, the recycling strategy is important.

- Allocation methods: we tested different allocation methods beyond the default of economic allocation. The MPW-BTX allocation factor for BTX (0.79) was changed to 0.69 (mass allocation), 0.46 (energy allocation), and 0.33 (economic allocation based on bio-oil prices). The biobased BTX allocation factor for BTX (0.59) was changed to 0.48 (mass), 0.46 (energy), and 0.16 (economic, bio-oil prices). “Bio-oil prices” refer to the market value of pyrolysis bio-oil, which is composed of light organics.^61^ The details are summarized in Supporting Information S1.3.
- Glycerol source: we considered glycerol production from other feedstock besides soybeans from the USA, including glycerol from rapeseed oil^62^ and palm oil,^63^ and soybeans cultivated at an another geographical location, i.e., Brazil. This was modeled by replacing the default glycerine data set with the following Ecoinvent 3.8 data sets: glycerine {BR}|esterification of soybean oil; glycerine {MY}|esterification of palm oil; and glycerine {Europe without Switzerland}|esterification of rape oil.
- Yields: the yields of the MPW- and biobased BTX production routes are uncertain. Based on expert judgment, we ranged the BTX yields from −10 to +20% compared to default. This affected (i) the amount of the BTX product and waste gases and thus also the supply of electricity that could be generated on site (CHP) and (ii) the allocation factors. The latter now ranged from 0.77 to 0.84 for MPW-BTX and from 0.55 to 0.7 for biobased BTX.
- Multiple electricity scenarios in 2050: alongside the SSP2 “middle of the road” baseline scenario of the electricity market of 2050, we tested a more optimistic pathway of 1.9 W/m^2^ (RCP1.9) as well as a more conservative pathway of 4.5 W/m^2^ (RCP4.5) in 2100.^43^ The baseline scenario represents efforts to commit to a long-term climate target of 2 °C, while RCP1.9 and RCP 4.5 include efforts resulting in an estimated global warming of up to 1.5 °C and up to 3.5 °C, in 2100, respectively.^43^
- Multiple recycling scenarios in 2050: alongside the baseline 2 °C-circular economy scenario, we tested less optimistic scenarios based on SSP2 RCP4.5 and a “worst case” narrative. The SSP2 RCP4.5 scenario included 14% chemical or mechanical recycling of plastics, 17% landfill stock, and 69% littered or incineration with energy recovery. The “worst case” scenario represented 100% littered or incinerated with energy recovery. The baseline 2 °C-circular economy scenario included 29% recycling, 58% landfill stock, and 13% littered or incinerated with energy recovery.^47^

### Resource Use Perspective

**Figure 2:**
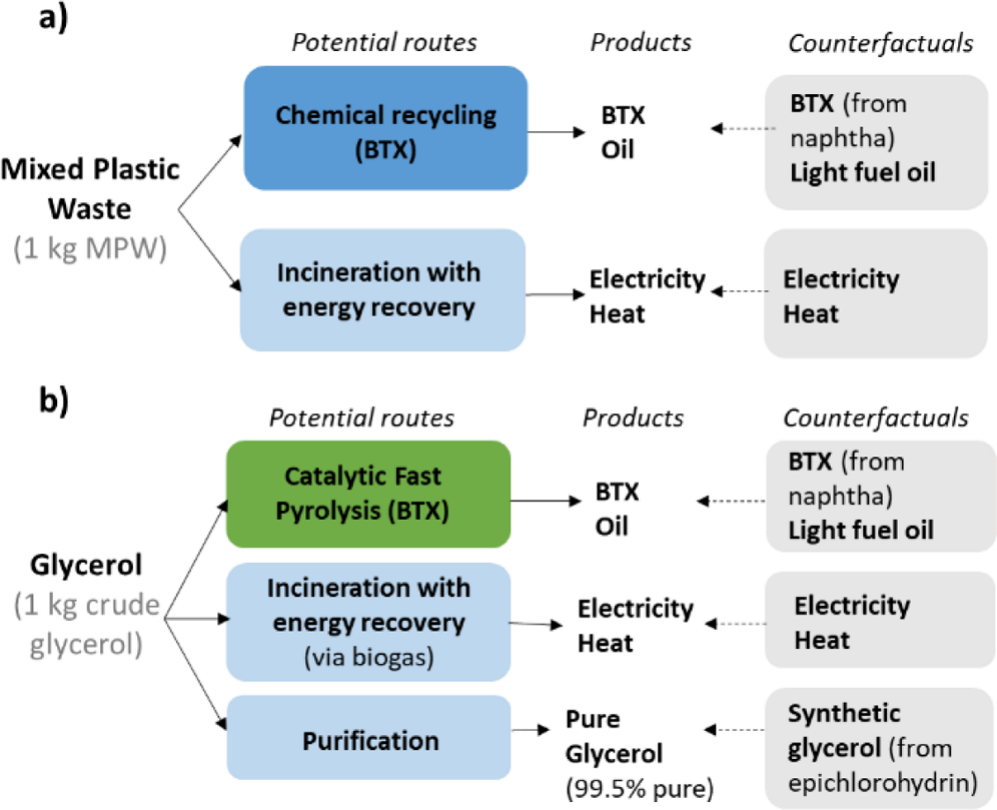
Representation of resource use perspective of (a) MPW and (b) glycerol as feedstock to produce BTX. The alternative uses producing other products (indicated by the arrow) are shown in blue, and the counterfactuals, i.e., the avoided products, are shown in the gray boxes. MPW = mixed plastic waste; BTX = benzene, toluene and xylene.

Biomass and mixed plastic waste can be used in a myriad of applications besides BTX production. To understand the relative merits of their use in BTX production, we assessed whether the production of BTX results in lower GHG emissions than other common applications of these feedstocks (Figure 2) following the approach by Hanssen and Huijbregts.^64^ For MPW (Figure 2a), the alternative application was incineration of plastic waste,^27^ with energy recovery based on average incineration efficiencies.^36^ Landfilling was excluded because the EU guidelines state: “landfilling is the least preferable option and should be limited to the necessary minimum”.^65^ For biomass (Figure 2b), the two alternative uses of glycerol considered were (i) combustion of biogas (fermented from glycerol)^37^ to generate electricity and heat,^27^ which we called “incineration with energy recovery”, and (ii) purification toward 99.5-grade glycerol.^38^ High-grade glycerol (99.5%) is alternatively still manufactured as synthetic glycerol, as medical and cosmetic applications need high quality glycerol.^66^ This was modeled via the process of synthetization of propylene via epichlorohydrin.^27^See Supporting Information S1.8 for further details. In this analysis, we accounted for the fact that biomass or MPW-based products would substitute conventional fossil products (counterfactuals indicated in gray boxes) and therefore resulted in avoided emissions that were quantified using Ecoinvent data.

## Results

### Mid- and Endpoint Impacts

**Figure 3:**
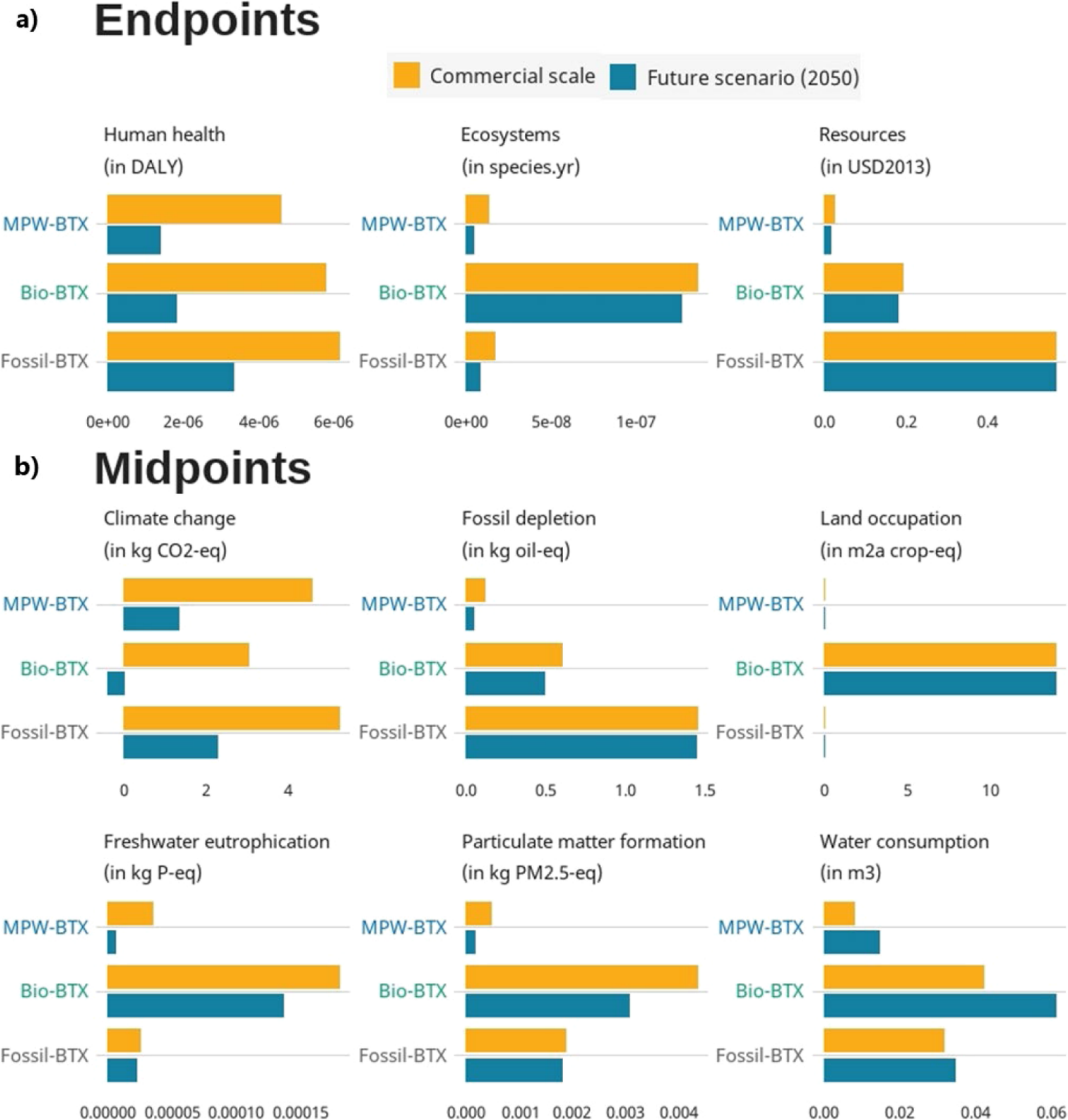
(a) Endpoint damage (exact numbers in Supporting Information S2.1), and (b) impacts of the six main contributing midpoint indicators of the commercial (2024) level and future, industrial (2050) level BTX production from MPW, glycerol, and fossil fuels. *Water consumption is contributing ∼1% to the end points ecosystems and human health, but it is the only impact category increasing in impact in the future and therefore shown here; the results of all midpoint impact categories are in Supporting Information S2.2.

**Figure 4:**
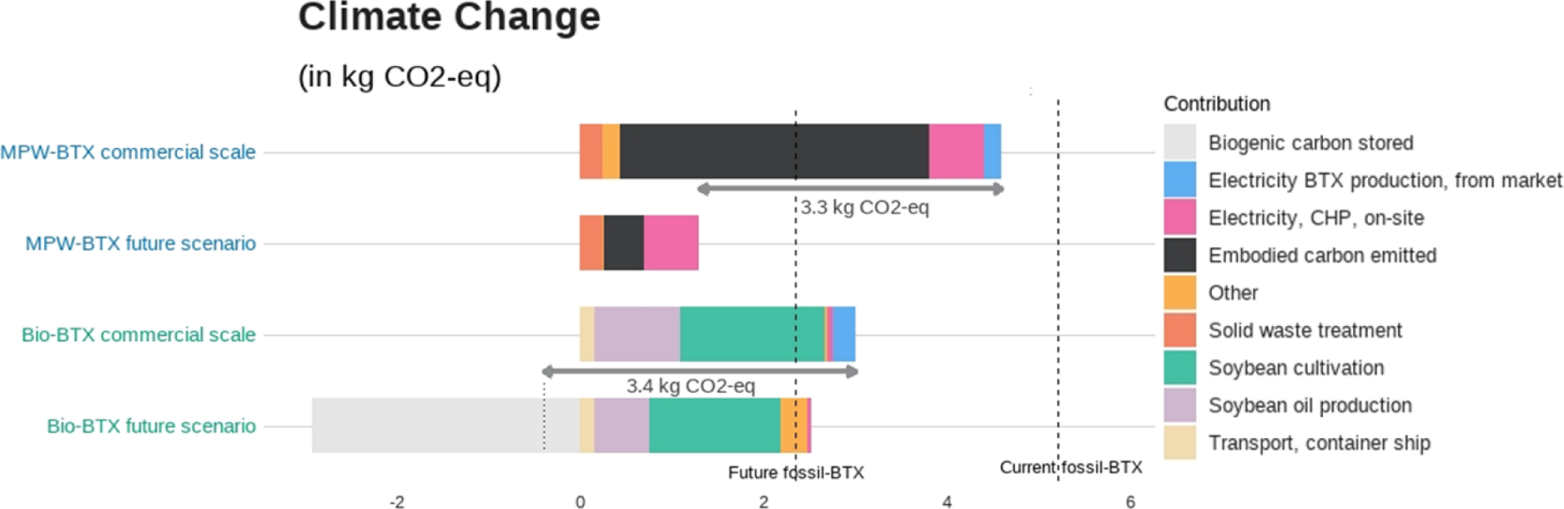
Process contributions to climate change impact of MPW, biomass, and fossil-based BTX production pathways. The difference between current and future production is indicated with the gray arrow. CHP = combined heat and power; MPW = mixed plastic waste.

Figure 3 shows the life-cycle impacts at endpoint (Figure 3a) and midpoint level (Figure 3b) of BTX produced from the different feedstocks. BTX from MPW resulted in the lowest potential impacts across the endpoint categories human health, ecosystems quality, and resource scarcity, compared to the other BTX pathways (Figure 3a). Nonetheless, both alternative pathways came with trade-offs on midpoint level (Figure 3b). MPW-BTX had the lowest predicted impact in all categories, except for climate change (and freshwater eutrophication, only at the commercial level and compared to fossil-BTX). Here, biobased BTX resulted in the lowest GHG emissions, i.e., 3.0 kg CO_2_-equiv per kg BTX (Figure 4), mainly due to its biogenic carbon content, which leads to carbon neutral end-of-life CO_2_ emissions. However, biobased BTX lead to higher impacts in multiple other midpoint categories: land occupation, fine particular matter formation, freshwater eutrophication, and water consumption. These higher impacts result from agricultural practices, i.e., the cultivation and harvest of soybeans for glycerol.

When applying the environmental footprint method at the midpoint level (Supporting Information S2.2), the same trends were observed; meaning, the biobased BTX pathway resulted in the lowest potential GHG emissions and fossil-BTX in the highest, while the MPW-BTX pathway had lower potential impacts across the other midpoints.

### Estimates of Future Life-Cycle Impacts

Figures 3 and 4 show that environmental impacts are likely to reduce in the future, with negative GHG emissions for biobased BTX (−0.4 kg CO_2_-equiv/kg BTX). This is mainly related to the end of life carbon flows integrated in the future scenarios, which avoids 87% of embodied carbon to be re-emitted.

Overall, largest future reductions were seen for MPW-BTX, with midpoint impacts decreasing with 15–85%. In contrast, biobased BTX impact reductions ranged up to 30% (with the exception of 113% for GHG emissions) and fossil-BTX impacts reduced up to 56%. In all cases, water consumption increased, varying between 9 and 83% (Figure 3b). This is caused by foreseen carbon capture and storage (CCS) in the future electricity market.

Apart from the effect of carbon recycling, the future reduction potential of fossil-BTX is relatively low because the electricity use in fossil-BTX production makes up only 1% of the total energy input, as it mainly depends on gas and oil. Moreover, the future GHG emission reduction potential of MPW-BTX was expected to be larger. Yet, the waste gases that are used for energy purposes on-site lead nevertheless to emissions due to the fossil carbon content of mixed plastic waste.

### Process Contributions to Climate Change Impact

GHG emission reductions ranged between 42 and 113% for biobased BTX and 12 and 71% for MPW-BTX (Figure 4), compared to current fossil BTX production. For the current commercial scenario, the largest contribution to climate change for both MPW- and fossil-BTX is related to the embodied carbon released in the form of CO_2_ at the end of life. The GHG emissions of MPW-BTX are mainly affected by the end of life treatment, rather than by the production process itself, which showed to be relatively low in GHG emissions. For biobased BTX, glycerol production contributed the most to climate change, and other midpoint categories, with 52% of it relating directly to soybean cultivation (Figure 4).

### Sensitivity Analysis

**Figure 5:**
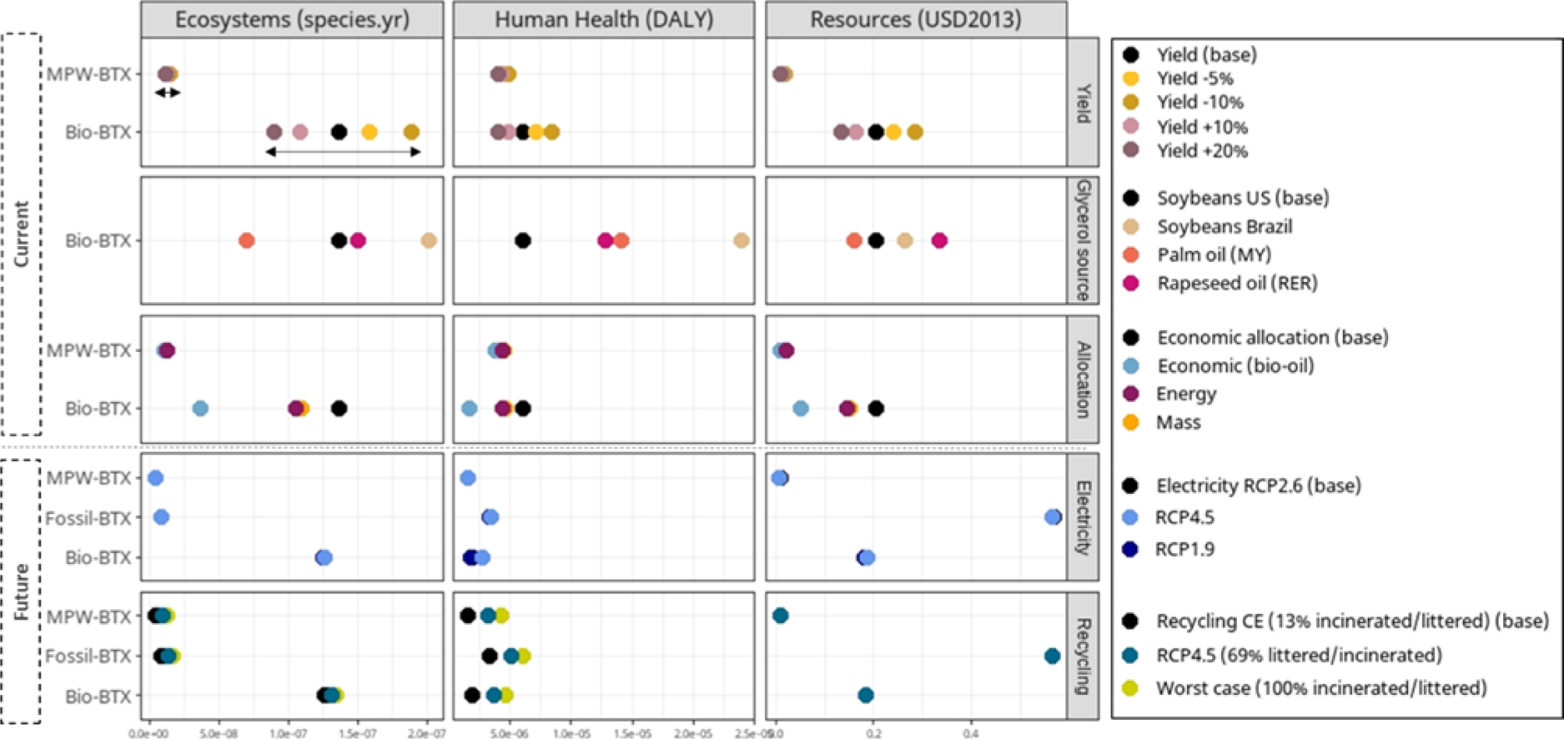
Sensitivity analysis results on an end point level, varying key modeling parameters and scenarios. MY = Malaysia, RER = Europe.

The type of allocation method influenced the environmental impact estimations of the BTX production pathways. Depending on either mass, energy, or economic allocation, the climate change impact of biobased BTX production ranged from 1.1 to 3.0 kg CO_2_-equiv/kg BTX for the current scenario, and that for MPW-BTX production ranged from 3.9 to 4.6 kg CO_2_-equiv/kg BTX (Supporting Information S2.7). The default scenario, economic allocation based on light fuel oil prices, led to results on the higher end of the ranges, while economic allocation based on bio-oil prices lead to the lowest results (Figure 5). Nevertheless, the general conclusions did not change depending on allocation method.

Glycerol production has a large influence on the end point results of biobased BTX (Figure 5). Producing glycerol with other feedstocks than soybeans from the USA led to potentially higher endpoint results, including GHG emissions. Largest GHG emissions result for glycerol from Brazilian soybeans or Malaysian palm oil, resulting in even 69–126% higher GHG emissions for biobased BTX compared to fossil-BTX. These higher predicted emissions were mainly due to clear-cutting of primary forest to arable land (Supporting Information S2.6). The environmental impact of biobased BTX thus highly depends on the location and production of glycerol and much less on the BTX production process itself.

The large impact of biomass input for biobased BTX is also identified by varying the parameter “yield” (Figure 5). This has a larger effect on biobased BTX than MPW-BTX because glycerol production has a relatively high impact, while plastic waste has no impact.

Depending on the future electricity scenarios, GHG emissions were lower ranging from 103 to 120% for biobased BTX and 34–46% for MPW-BTX, compared to future fossil-BTX production (Supporting Information S2.4; Figure 5 for endpoint results). Future BTX production including electrification of the processes and a renewable energy mix can thus reduce impact on the end point level and mainly climate change impact.

The influence of carbon recycling on the results was further shown by testing alternative plastic recycling scenarios for 2050, which resulted in GHG emissions of −0.4 to 1.6 kg CO_2_-equiv/kg BTX for biobased BTX, 1.3 to 3.2 kg CO_2_-equiv/kg BTX for MPW-BTX, and 2.3 to 4.1 kg CO_2_-equiv/kg BTX for fossil-BTX (Supporting Information S2.5). The alternative recycling strategies increase the impact in the endpoint categories Ecosystems and Human Health (Figure 5). For biobased BTX, combining biomass use with plastic recycling could lead to a net carbon sink.

### Planetary Boundary Impacts

**Figure 6:**
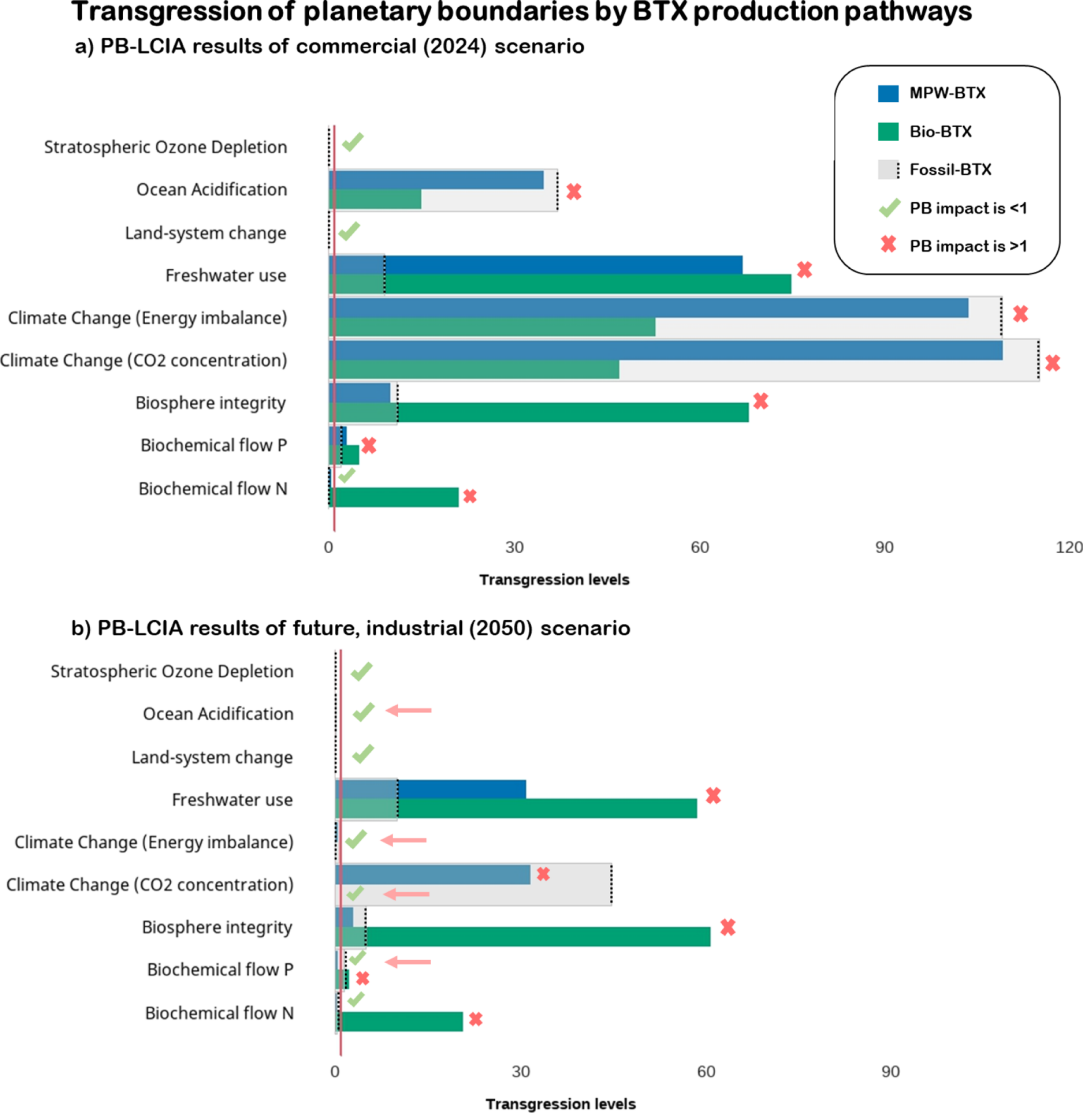
Transgression of planetary boundaries by BTX production pathways. (a) Commercial (2024) scenario and (b) future, industrial scenario (2050, as described in section “Estimates of Future Life-Cycle Impacts”). The green check marks indicate that the PB-LCIA result is <1. The red crosses indicate that the results are >1, and thus, the BTX pathway is transgressing its share of safe operating space of that planetary boundary. The pink arrow indicates reductions in transgression levels to <1. BTX = benzene, toluene and xylene, MPW = mixed plastic waste. Exact numbers are given in Supporting Information S2.3.

The results from the PB-LCIA are presented in Figure 6a for the commercial (2024) scenario and in Figure 6b for the future (2050) scenario. If the transgression level is >1, the BTX pathway overshoots the safe operating space that was allocated to BTX production. Only when all the transgression levels are <1, BTX production is predicted to be “absolutely” sustainable. At the current commercial scale (Figure 6a), all BTX pathways transgressed at least six levels of the planetary boundaries, meaning none of the pathways are considered sustainable in absolute terms. The BTX pathways in the future scenario (Figure 6b) lead to the same conclusion, albeit that only three levels of the planetary boundaries were transgressed.

The climate change levels were transgressed up to 115 times, but the least by biobased BTX production due to its biogenic carbon content. Consequently, all pathways transgressed the levels of ocean acidification and biosphere integrity, as they are strongly affected by CO_2_ emissions. In the future scenario, especially climate change (energy imbalance) and ocean acidification were affected due to carbon recycling, leading to transgression levels of <1. Furthermore, the biosphere integrity and biochemical N and P flow levels were specifically high for the biobased BTX pathway. Especially agricultural practices and land use related to soybean cultivation increased the impact of biobased BTX.

The application of the carrying capacity normalization factors to the EF results also identified climate change as the highest impact category for all BTX pathways, as well as ecotoxicity and land use for biobased BTX. Interestingly, the normalization-method ranked particulate matter high in all BTX pathways. This category is related to atmospheric aerosol loading, which is not yet adequately defined and therefore excluded in this PB-LCIA assessment.

### Optimal Use of Resources

**Figure 7:**
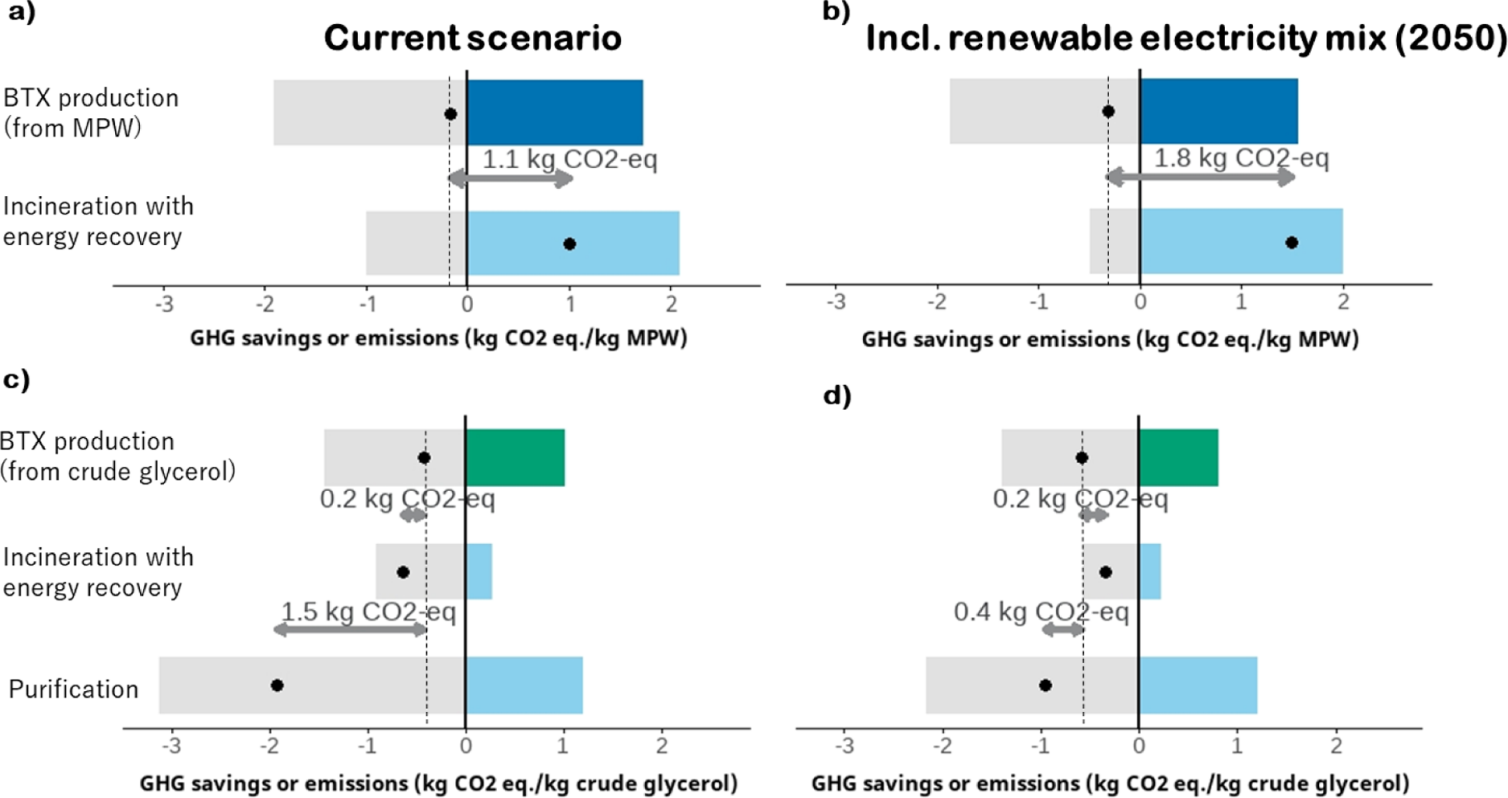
Climate change impact and savings for the use of (a,b) 1 kg of MPW and (c,d) 1 kg of crude glycerol, at the current commercial scale and including a future renewable electricity mix (SSP2-RCP2.6, 2050). The black dot represents the GHG emissions minus the GHG saving potential. The arrow indicates the GHG benefit/disadvantage of BTX production compared to the other uses. BTX = benzene, toluene and xylene; MPW = mixed plastic waste.

Using MPW to produce BTX instead of incinerating it and recovering energy resulted in a GHG benefit of 1.1 kg CO_2_-equiv/kg mix plastic waste used (Figure 7a), mainly because incinerating plastic waste emits large amounts of CO_2_. Figure 7b shows that the relative climate benefits of using MPW for BTX increases to 1.8 kg CO_2_-equiv/kg feedstock applying a 2050-projected renewable electricity mix. The main reason for this increase is that the GHG savings of energy recovery from incineration diminish in the future, as an increasingly cleaner electricity mix is substituted.

Figure 7c,d shows that incineration with energy recovery or higher-grade glycerol has higher GHG benefits compared to BTX production. Here as well, the relative climate benefit for incineration with energy recovery is expected to decrease in the future due to a cleaner energy mix. For purification, the GHG benefit relates to the avoided conventional production of synthetic glycerol which is a GHG intensive process.

## Discussion and Conclusions

### Environmental Impacts

This is the first study to compare the environmental impacts of BTX production using three different carbon feedstocks. The environmental impacts (midpoint and endpoint) were the lowest for future MPW-BTX, except for GHG emissions. Nevertheless, from a resource use perspective, MPW-BTX was favorable over waste incineration with energy recovery with a GHG benefit of 1.1 kg CO_2_ equiv/kg plastic waste, whereas using glycerol for BTX production resulted in a GHG disadvantage compared to other uses. These findings show the added value of multiple perspectives within performing an LCA.

Our results highlight the importance of including impacts beyond GHG emissions in environmental impact analyses and show the environmental trade-offs between the various feedstocks. In general, these trade-offs result from agricultural practices like fertilization and pesticide use that can increase eutrophication, acidification, and ecotoxicity.^67^

We excluded land use change (LUC)-related emissions for glycerol production. Even though this is fair practice,^68^ GHG emissions from LUC can play a big role with first generation biomass or when deforestation is involved.^69−71^ This was also shown in the sensitivity analysis on different feedstocks for glycerol production, where impacts were predicted to be higher compared to soybean cultivation in the USA mainly as a result of clearing the original vegetation, and in some cases, they even resulted in higher overall emissions compared to fossil-BTX. Moreover, LUC emissions can also encompass soil carbon losses and lost capacity of natural vegetation to sequester CO_2_.^72,73^ These were not included due to modeling limitations implying that the GHG emissions of biobased BTX may be underestimated.

### Climate Change Impacts

We found that especially the process-related emissions from production were low compared to fossil-BTX. According to literature on other BTX production pathways in development, process-related CO_2_ emissions are predicted at 2.21 kg CO_2_-equiv/kg BTX for a Diels–Alder route and 2.6 kg CO_2_-equiv/kg BTX for a methanol-to-aromatics route.^41^ The current GHG emissions related to MPW- and biobased BTX processing were estimated in the range between 0.7 and 1.3 kg CO_2_-equiv/kg BTX. This shows that the catalytic fast pyrolysis process has potential over these alternative routes.

There is a GHG benefit to treat MPW via chemical recycling to produce BTX. The results from this study’s resource use perspective are in line with the previous research on chemical recycling versus incineration with energy recovery.^16,17,29^ In accordance with this study, a GHG benefit of 1.1 kg CO_2_-equiv/kg waste feedstock was treated to produce BTX, van der Hulst et al.,^17^ estimated a GHG benefit of 0.82 kg CO_2_-equiv/kg waste feedstock treated for chemical recycling producing high value chemicals, and a 50% lower climate change impact for chemical recycling via pyrolysis was found by Jeswani et al.^29^ Even though direct comparison is not possible because different fossil-based chemicals are avoided, i.e., BTX, other high value chemicals or naphtha, these studies uniformly show emission saving potentials for chemical recycling when compared to incineration with energy recovery.

We did not find a GHG benefit to treat glycerol to produce BTX: purification of glycerol was the better option due the avoided conventional production of synthetic glycerol which is a GHG intensive process, which has become economically feasible.^66^ Moreover, producing electricity from biogas is currently promoted in European renewable energy policies, because it displaces the use of fossil fuels in energy supply and contributes to GHG emission reductions,^74^ which makes glycerol as the feedstock choice for BTX less logical. Due to limited data, the resource use perspective included GHG emissions only. Expanding the analysis to other environmental impacts, however, could generate further insights into the beneficial purposes of the feedstocks.

We found positive emissions for current biobased BTX production of 3 kg CO_2_-equiv/kg BTX, while Yang et al.^14^ found negative emissions of 0.82 kg CO_2_-equiv/kg biobased BTX. The lower impact was mainly a result of the carbon credits from exported electricity which offset upstream emissions, i.e., substitution. In this study, if the byproducts, i.e., bio-oil and the surplus of electricity, were substituted, this would result in a credit of 2.25 kg CO_2_-equiv/kg biobased BTX (Supporting Information S3.3). Taking this credit into account, the GHG emissions of this study’s biobased BTX are nevertheless still higher due to the high impact of soybean cultivation. In both cases, however, the credits would diminish toward 2050 if we assume electricity will be renewably produced. This highlights the added value of a future assessment.

The largest share of emissions of biobased BTX originated from glycerol production. Lower GHG emissions in Yang et al.’s work were also a result of the feedstock selection of wood chips.^14^ In line with this, lower climate impacts were also found for pulpwood as a feedstock in an intermediate biobased BTX production.^10,76^ Due to limited process and technology data, we did not further research woody biomass as a feedstock.

In the future scenario, we applied the default economic allocation ratio, which is based on the average of 2011–2021 prices. Ideally, as economic allocation reflects socio-economic demands, future pricing was considered in the 2050 scenario. However, there is a large uncertainty regarding price forecasting, as it depends on many factors, such as fluctuations, policy and technology development.^75^

For both fossil and MPW-BTX, a large share of their climate change impact related to the embodied carbon released at the end of life. End-of-life emissions are, however, often not included in petrochemical GHG emissions reporting.^77,78^ In our current commercial (2024) scenario, it was assumed that all carbon embodied in the products would eventually end up in the atmosphere. Large reductions in the future scenario were, therefore, mainly a result of continuous carbon recycling, avoiding 87% of the embodied carbon to be emitted. Preventing the end products, for which BTX is used, from being burned or incinerated for energy is thus pivotal in reducing the environmental impact of both fossil as well as renewable BTX production.

### Absolute Sustainability

Even though the alternative BTX pathways showed lower environmental impacts compared to fossil-BTX pathway, at least three planetary boundaries were transgressed. Tulus et al. found that most of 492 globally produced chemicals transgress multiple planetary boundaries.^58^ A study on the petrochemical industry replacing fossil feedstock with carbon via carbon capture and utilization (CCU) technologies demonstrated emission reductions from 25% up to 100%, though in the best case it still exceeded biosphere integrity.^4^ These and our findings highlight the relevance of complementing LCA with an absolute environmental sustainability assessment to further support decision making toward the development of environmental sustainable production chains. LCIA helped us to understand what the hotspots in the BTX production chain were, while the PB-LCIA showed that further reduction is still necessary to stay within the planetary boundaries.

The share of safe operating space depends on downscaling of the safe operating space; it can thus vary per study and has a large influence on the results. Here, we used the transgression levels defined by Tulus et al.^58^ based on equality and the economic value of 2018. Whether a more expensive product is allowed to take up more safe operating space is in the end of a political question, and ideally different downscaling perspectives are therefore considered. In general, downscaling of planetary boundaries is still in its infancy, and future research should be dedicated exploring alternative definitions of transgressions levels.

### Recommendations for a Sustainable Future of BTX Production

For both alternative BTX pathways, the feedstock choice has a large influence on the environmental impacts, i.e., the fossil carbon content in plastic waste for MPW-BTX and biomass cultivation for biobased BTX. Therefore, to further reduce environmental impacts of the MPW-BTX production, the GHG emissions related to the embodied carbon at end-of-life should be further avoided by reusing and recycling plastics and other products where BTX is used in. If 100% of the embodied carbon remains in the system, GHG emissions could be 0.86 kg CO_2_-equiv/kg MPW-BTX, i.e., 83% lower than current fossil BTX production. Furthermore, the emissions related to the on-site electricity production from the waste gases could be abated by, for example, CCS or CCU technologies. Theoretically, this could save a further 0.6 kg CO_2_-equiv/kg BTX leading to 0.26 kg CO_2_-equiv/kg MPW-BTX, though this excludes the environmental impacts of CCS and CCU.^14,79^ Alternatively, it might be possible to use the waste gases as feedstock for other production, such as methanol, to keep the carbon in the loop.^80^

Increasing the share of biogenic carbon content in plastics could further reduce the GHG impact of MPW-BTX. If 45% of the mixed plastic waste would be sourced from biomass, future MPW-BTX could decrease to −0.4 kg CO_2_-equiv (Supporting Information S3.2), comparable to future biobased BTX’s impact. Chemical recycling of biobased plastics could thus combine the benefit of biogenic carbon with carbon recycling, which could result in long-term CO_2_ sequestration from the atmosphere.^47^ This would, however, require the use of sustainably sourced biomass and further exploration of the potential-associated trade-offs with other environmental impacts.

To further reduce environmental impacts of the biobased BTX production, other biobased feedstocks could be considered. In general, research showed that the use of woody biomass or agricultural residues, such as sugar cane bagasse or corn stover, can lead to lower GHG emissions, eutrophication, and land use impacts than the use of first generation biomass.^81−83^ The use of these feedstocks could lower the GHG emissions of the BTX’s feedstock phase by 74–95%, compared to soybean glycerol (Supporting Information S3.2).^84−86^ When residue biomass is considered to have no environmental impact, i.e., “zero-burden approach”,^87^ it would lower the GHG impact of biobased BTX production with at least 1.4 kg CO_2_-equiv/kg BTX. Further development of low impact lignocellulose-based BTX production to a commercial scale would therefore be recommended.

In view of the feedstock supply, there are factors of influence that should be further researched to support policy recommendations. In regards to glycerol, there is pressure from competing technologies for renewable diesel, which do not produce glycerol as a byproduct;^66^ plus, glycerol has a relatively high price. Moreover, there are many other glycerol applications being developed or promoted that might have larger environmental benefits.^74,88^ Plastic waste, on the other hand, has a GHG benefit compared to incineration with energy recovery and is abundant. Either based on current plastic waste management trends^89^ or a middle-of-the-road development scenario,^47^ by 2050, 40–58% of the generated plastic waste would be required to meet BTX demands (Supporting Information S3.3). However, there could be “competition” with mechanical recycling to retrieve plastics or chemical recycling producing other high value chemicals and fuels.^18,90^ Moreover, policy actions targeting plastic use, such as reducing single-use plastics,^91^ may result in lower amounts of feedstock availability. Hence, future studies to assess holistically the cost-benefit and trade-offs at a macro scale of the different choices will be necessary.

A combination of strategies proves to be key to reach a low-emission industry. Our findings imply that the use of alternative carbon feedstock, electrification of the processes and a renewable electricity mix could reduce emissions of BTX production up to 21–58% in 2050, compared to fossil BTX production. Including carbon recycling of 87% can reduce GHG emissions even up to 75–107% by 2050. In contrast, solely decarbonizing energy supply reduces GHG emissions by 8–20%. To further minimize emissions, recycling and/or CCS technologies could be used to abate end-of-life and process emissions.^92^

Overall, the combination of methods applied in our research offered complementary insights into the sustainability of the alternative BTX pathways. In the context of the safety and sustainability by design recommendations,^19^ combining an LCA and absolute sustainability assessment gives insights into whether one product design is more sustainable than the other and whether it stays within the planetary boundaries. Overall, more systemic changes would be necessary for BTX production to stay within the planetary boundaries, such as the use of other types of waste biomass, increasing carbon recycling, and the abatement of end-of-life impacts, alongside reducing product demand.^92,93^ To conclude, future BTX production combining strategies including alternative carbon feedstock helps the petrochemical industry to become more sustainable. Holistic assessments similar to the one presented herein can guide the research and policy in their support to develop more sustainable aromatics and other petrochemicals.

## Data Availability

All relevant data supporting the findings of this study are available within the article and its Supporting Information files.

## Supporting Information Available

System description of bioBTX, carbon accounting, allocation strategies, calculation of distillation step, framework for the evaluation of emerging technologies, electricity use in fossil-BTX production, description of PB-LCIA impact assessment, and resource use perspective; all mid- and endpoint results, absolute assessment results, sensitivity analyses, and uncertainty analysis; and additional calculations on other types of biomass feedstock and plastic feedstock supply (PDF)
