# Supplementary material for: Evaluating the Environmental Sustainability of Alternative Ways to Produce Benzene, Toluene, and Xylene
Source: ACS Sustain Chem Eng. 2024 Mar 18;12(13):5092–104. doi: 10.1021/acssuschemeng.3c06996 (PMC10988839; doi:10.1021/acssuschemeng.3c06996)
Supplement: Supplementary file 1 — sc3c06996_si_001.pdf [file sc3c06996_si_001.pdf]

Supplementary Information

Evaluating the environmental sustainability of alternative ways to produce benzene, toluene and xylene

Emma A. R. Zuiderveen* [1,2], C. Caldeira[2,5], T. Vries[3], N.J. Schenk[3], M.A.J. Huijbregts[1,4], S. Sala[2], S.V. Hanssen[1], R. van Zelm[1]

[1] Department of Environmental Science, Radboud Institute for Biological & Environmental Sciences, Radboud University, Nijmegen, the Netherlands
[2] European Commission, Joint Research Centre, Via Enrico Fermi 2749, 21027, Ispra, VA, Italy
[3] BioBTX, Zernikelaan 17, 9747 AA, Groningen, The Netherlands
[4] Department of Circularity & Sustainability Impacts, TNO, Princetonlaan 6, 3584CB Utrecht, The Netherlands
[5] Syensqo Lyon Research and Innovation Center, 85 Avenue des Freres Perret 69190 Saint-Fons, France
* Corresponding author: emma.zuiderveen@ru.nl

S1 Materials & methods 2
S1.1 BioBTX 2
S1.2 Carbon accounting 2
S1.3 Allocation 2
S1.4 Distillation 4
S1.5 Evaluating emerging technologies 4
S1.6 Electricity use in fossil-BTX pathway 5
S1.7 Impact assessment PB-LCIA 5
S1.8 Resource use perspective 7
S2 Results 10
S2.1 Endpoint results 10
S2.2 Midpoint results 10
S2.3 Absolute assessments results 12
S2.4 Sensitivity Analysis: electricity market scenarios for 2050 13
S2.5 Sensitivity Analysis: plastic recycling scenarios for 2050 13
S2.6 Sensitivity Analysis: Glycerol production from other feedstock 14
S2.7 Sensitivity Analysis: Allocation strategies 15
S3 Discussion 17
S3.1 Impact other types of biomass feedstock 17
S3.2 Plastic feedstock supply 17
S3.3 Substitution 18

S1 Materials & methods

S1.1 BioBTX

The BTX production pathways (Figure 1 in the main text) from both mixed plastic waste and biomass is based on the Integrated Cascading Catalytic Pyrolysis process developed by BioBTX, a pilot-company located in the Netherlands. Firstly, the feedstock is the thermally cracked; secondly, the pyrolysis vapours are catalytically converted to BTX; thirdly, the BTX is separated and collected. BioBTX' primary data is based on a capacity of 48 kt feedstock/year and includes the core technology and downstream steps[1].

S1.2 Carbon accounting

The system boundary was set to cradle-to-grave with an incineration end-of-life scenario based on the chemical structure of BTX. The fossil carbon content can therefore lead to an additional 3.36 kg CO_2 eq./kg BTX (Table S.1). In the case of bio-BTX, the additional CO_2 emissions were considered neutral. This was justified following the biogenic global warming potential (GWP_{bio}) approach by Cherubini et al.[3] and allocating temporary carbon storage in bio-products based on Guest et al.,[5], where biogenic carbon can be considered neutral, because both the crop rotation period and the storage period in the technosphere are short. This method accounts for the fate of the carbon embedded in the end products.

Table S1: data on carbon content BTX and related emissions

	Chemical formula	Molar weight (g/mol)	CO_2 emissions related to carbon content
**Benzene**	C_6H_6	78.11	3.38 kg CO_2
**Toluene**	C_7H_8	92.14	3.35 kg CO_2
**Xylene**	C_8H_{10}	106.16	3.32g CO_2

S1.3 Allocation

Figure S.1 indicates the points of allocation in the MPW- and bio-BTX pathways. Table S.2 is an overview of the different allocation factors including calculations.

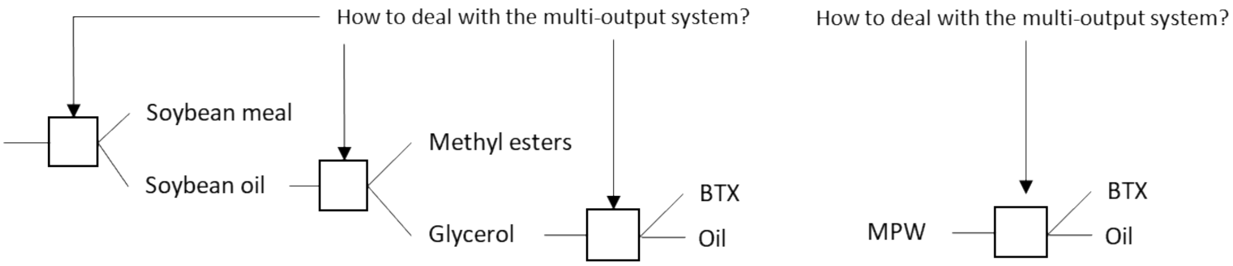

Figure S1: overview of points of allocation in both MPW- and bio-BTX pathways.

Table S2: allocation factors for mass, economic and energy allocation. Fossil-BTX production is based on economic allocation only, due to the aggregated dataset in Ecoinvent, see 1.1.6 for more information. MPW = Mixed Plastic Waste; LHV = Lower Heating Value; BTX = benzene-toluene-xylene.

	Products	Calculation	Allocation factor
Mass allocation	MPW BTX	m_p / m_t	BTX: 0.69<br>Oil: 0.31
	Bio BTX	m_p / m_t	Meal: 0.19<br>Oil: 0.81<br><br>Methyl esters: 0.90<br>Glycerol: 0.10<br><br>BTX: 0.48<br>Oil: 0.52
Energy allocation	MPW BTX	m_{px} LHV / ($m_{p1,2,3}$ $LHV_{p1,2,3}$)<br>LHV BTX: 40.5<br>LHV natural gas: 47.1<br>LHV light fuel oil: 43	BTX: 0.67<br>Oil: 0.33
	Bio BTX	m_p LHV / ($m_{p1,2}$ $LHV_{p1,2}$)<br>LHV BTX: 40.5<br>LHV methyl esters (biodiesel): 43.5 MJ/kg<br>LHV glycerol: 18 MJ/kg<br>LHV Soybean meal: 15.4 MJ/kg<br>LHV Soybean oil: 37 MJ/kg	Meal: 0.64<br>Oil: 0.36<br><br>Methyl esters: 0.96<br>glycerol: 0.04<br><br>BTX: 0.46<br>Oil: 0.54
Economic allocation	MPW BTX	m_p $Price_p$ / ($m_{p1,2,3}$ $Price_{p1,2,3}$), prices taken from 2011-2024 with the exception of bio-oil, data was available for 2011, 2018, 2019, 2020.<br>1. Bio-oil price<br>2. Bio-oil as light fuel oil price	1. BTX: 0.33<br>Oil: 0.67<br><br>2. BTX: 0.79<br>Oil: 0.21
	Bio BTX	m_p $Price_p$ / ($m_{p1,2,3}$ $Price_{p1,2,3}$), prices taken from 2011-2024 with the exception of bio-oil, data was available for 2011, 2018, 2019, 2020.<br>1. Bio-oil price<br>2. Bio-oil as light fuel oil price<br>m_p $Price_p$ / ($m_{p1,2}$ $Price_{p1,2}$) for glycerol and methyl esters (2011-2021)	Soybean oil: 0.34<br>Soybean meal: 0.66<br><br>Methyl esters: 0.93<br>Glycerol: 0.07<br><br>1. BTX: 0.16<br>Oil: 0.84<br><br>2. BTX: 0.59<br>Oil: 0.41

S1.4 Distillation

An additional distillation step is applied to the crude BTX output, based on a simplified distillation step. Calculations are based on Piccinno et al., (2016), sum over each of the three chemicals is taken:

$$Q_{distillation} = \frac{C_p * m_{mix} * (T_{boil} - T_0) + \Delta H_{vap} * m_{dist} * (1.2 * R_{min} + 1)}{\eta_{heat} - 0.1} \quad (S1)$$

where C_p is the specific heat capacity, m_{mix} and m_{dist} the mass of the mixture and distillate, T_{boil} the boiling point and T_0 the room temperature, ΔH_{vap} the enthalpy of evaporation, R_{min} the minimum reflux ratio (set to 1, due to lack of information), and η_{heat} the heating efficiency is, heating losses are assumed to be 31% [6].

S1.5 Evaluating emerging technologies

Here, we follow the framework of van der Hulst et al.,[7] based on three successive steps to evaluate technology maturing up to industrial scale. The upscaling involves both technical aspects as well as adjustments to background processes. The steps are shown in Table S.3.

Table S3: stepwise approach for technology maturing. TRL = Technology Readiness level; MPW = Mixed Plastic Waste; ICCP = Integrated Cascading Catalytic Pyrolysis; SSP = Shared Socioeconomic pathway.

**Steps**	**Modelled changes**	**Current scale (2024)**	**Commercial scale (base scenario; 2024)**	**Industrial scale (2050)**	**Fossil-based production (2024, 2050)**
**Phase I**: definition TRL stage	Technical Development	TRL 7	TRL 9	TRL 9	TRL 9
	Feedstock	Biomass, MPW	Biomass, MPW	Biomass, MPW	Oil, naphtha
	Technology	ICCP	ICCP	ICCP	Catalytic reformate & steam cracking
**Phase II**: process changes, size scaling and process synergies	Process changes	-	Including downstream steps, increased yield and energy input	Increased energy efficiency based on a generic energy reduction rate of 1% per year[9–11]	-
	Product scaling	0.8 kton/year	48 kton/year	Since the FU is formulated as "1 kg of BTX production" and assuming linear size scaling, interventions remain the same.	-
	Process synergies: *Heat recovery & Reduction of waste streams*	-	An on-site gas system is proposed producing electricity with waste gasses: • A boiler with $Elec_{eff}$ of 28% is applied[8]	An on-site gas system is proposed producing electricity with co-produced gasses: • A boiler with $Elec_{eff}$ of 28% is applied[8]	-

**Phase III**: industrial learning and external developments	Changes to background processes	-	Electricity market of 2020[12]	Electricity market of 2050[13], based on narrative: • SSP2 RCP2.6 (baseline)<br>• SSP2 RCP4.5<br>• SSP2 RCP1.9	Same as industrial scale BTX production
		-	End of life processing.<br>• C-loss: burned or waste to energy: 100%	Plastic flows, SSP2 RCP 2.6 baseline scenario for 2050 [14]<br>• C-loss: Burned or waste to energy: 13%<br>• C-stock: Landfill, chemical and mechanical recycling: 87%	Same as industrial scale BTX production

S1.6 Electricity use in fossil-BTX pathway

Each of the separate Ecoinvent 3.8 datasets on naphtha-based benzene, toluene and xylene are formatted as a unit process, therefore "electricity" as input from the technosphere is not indicated as such. For this reason, the electricity use could not be automatically adapted to the future electricity market dataset. Table S.4 includes the total electricity for fossil-BTX which was separately assessed.

Table S4: total electricity use in fossil-BTX process, to adapt to a electricity market of 2050. Data on primary energy and share of electricity is taken from the original PlasticsEurope data documentation[15]*.*

	**Primary energy (MJ)**	**Share electricity of total energy demand (%)**	**Share mass (%)**	**Total electricity in MJ per kg BTX**
**Benzene**	80.3	0.5	48	0.19
**Toluene**	65.7	0.3	33	0.07
**Xylenes**	67.6	1	19	0.13

S1.7 Impact assessment PB-LCIA

In the following section the PB-LCIA impact assessment is discussed step by step, with a summary in Table S.5. The LCI elementary flows are converted into impacts on the Safe Operating Spaces (SOS) of the Planetary Boundaries (PB) by multiplying the flows with characterization factors (Equation S2) taken from Ryberg et al.[16]. For the missing CFs on biosphere integrity, the approach of Galán-Martín et al.[17] was adopted.

The transgression level is defined as the impact of the product in relation to the safe operating space of the PB (Equation S3). This involves downscaling, i.e. allocating, the safe operating space to the level of

BTX production (Equation S4). This is done based on the sharing principle 'equal per capita', assuming it is fair to share equally among the population. To then translate one's personal share of SOS to the chemical BTX, allocation was based on economic value. This based on the assumption that a higher economic value promotes well-being and therefore should result in a larger share of the SOS. For more explanation on the transgression level definitions, read Tulus et al.[18].

Furthermore, the life cycle impact assessment requires a continuous input, because PBs are expressed in annual threshold levels[19]. Therefore, the functional unit should include a time dimension (kg/year)[20]. In our case this is defined as total annual BTX production (in weight). However, this cancels out when combining equation S3 and S4.

Equation S2 $$\mathrm{IMP}_{\mathrm{b,\,BTX}} = \sum LCI_{e,\,BTX}\, CF_{eb}$$

Equation S3 $$\mathrm{TL}_{\mathrm{b,\,BTX}} = \frac{IMP_{b,\,BTX}}{sos_{b,\,BTX}}$$

Equation S4 Based on equal per capita approach and gross value added:

$$\mathrm{sos}_{\mathrm{b,\,BTX}} = \frac{SOS_b}{pop^{TOT}} pop_{btx} \frac{GVA_{BTX}}{GVA^{TOT}}$$

Combining S3 and S4 gives:

Equation S5 $$\mathrm{TL}_{\mathrm{b,\,BTX}} = \frac{IMP_{b,\,BTX}\, GVA^{TOT}}{SOS_b\, price_c}$$

$LCI_{e,BTX}$ is a LCI elementary flow e in BTX production, CF_{eb} is the characterization factor for the LCI elementary flow *e* linked to PB *b* (for an overview of the PBs, see Table S.3). The characterization factors are taken from Ryberg et al.[16,21], see Supplementary-file S.2. The pop^{tot} represents the total population and pop_{btx} the population that benefits from BTX production, which was assumed equal in this case. The GVA_{BTX} is the gross value added associated with BTX, GVA^{TOT} is the total gross value added of the world.

Table S5: AESA methods and definitions, summary of Planetary Boundary concepts and the PB-LCIA method.

Planetary Boundary (PB)	Control Variable (CV)	Safe Operating Space (SOS)
Climate change	Atmospheric CO2 concentration (ppm)<br>Energy imbalance at top-of-atmosphere (W m^{-2})	72<br>1
Stratospheric ozone depletion	Stratospheric O3 concentration (DU)	15
Ocean acidification	Carbonate ion concentration (Ωarag)	0.69
Biogeochemical flows	Global: P flow from freshwater to ocean (Tg P yr^{-1})<br>Global: biological fixation of N (Tg N yr^{-1})	6.6<br>62
Land-system change	Global: area (%) of original forest cover	25
Freshwater use	Global: maximum consumptive blue water use ($km^3 yr^{-1}$)	4000
Biosphere integrity	Biodiversity intactness (% BII loss)	10
**PB-LCIA**		

Method	Characterization-based: CFs linking LCIA to PBs [16,21]. Additionally, for biosphere integrity, CF is based on the approach of Galán-Martín et al. (2021), build on work from Hanafiah et al.[22] and updated using Wilting et al.[23] and GLOBIO 3.5 MSA values[24].
Sharing Principle	Equal per capita (EPC) and gross value added (GVA)
Equation PB-LCIA	$IMP_{pb,BTX} = \sum_{e \in E} LCI_{eBTX} CF_{epb}\ ,\ \forall pb\ \in PB,\ (S6)$ *where $IMP_{pb,BTX}$ is the impact of the production of BTX (per FU) related to the planetary boundary (pb), where PB is the total of all the planetary boundaries, LCI_{eBTX} is the life cycle inventory elementary flow e (expressed over time) associated with BTX production, CF_{epb} is the characterization factor (CF) that maps the elementary flow e onto the control variable.*
Equation transgression level	$TL_{pb,\ BTX} = \frac{IMP_{pb,\ BTX}\ GVA^{TOT}}{SOS_{pb}\ price_c}\quad (S5)$ *where $IMP_{pb,BTX}$ is the LCI impact mapped onto the control variable (CV) of planetary boundary pb. The GVA^{TOT} is the total gross value added of the world - the global GVA is retrieved from The World Bank database. To be consistent, the basic prices ($price_c$) are taken from Tulus et al. (2021): 2.55 (xylene), 0.73 (toluene) and 0.88 (benzene) in USD2018. The SOS_{pb} is the total SOS for CV_{pb}. For the steps to arrive at this equation, see Supplementary S.10 and the work of Tulus et al., 2021.*

S1.8 Resource use perspective

Mixed plastic waste

Currently, MPW is most commonly incinerated with energy recovery[25]. To model the energy recovery from incinerating mixed plastic waste, the PEF guidelines[26] are followed (S7). An average Dutch incineration efficiency is assumed: 33.3%, with 28% for electricity and 9.3% thermal efficiency, and 4% of electricity for self-consumption[27]. Only electricity is considered in our model. Nevertheless, the heat generated could be further used leading to additional benefits. To account for the avoided product, i.e. bio-oil, light fuel oil is used. The lower heating value from different sources are shown in Table S.6. Based on the Product Environmental Footprint (PEF) the following energy recovery formula is used:

$$E_{recovery} = E_{ER} - LHV \times E_{se,heat} \times X_{ER,heat} - LHV \times E_{se,elec} \times X_{ER,elec} \qquad (S7)$$

Where $E_{recovery}$ is the energy recovery with credits for avoided primary energy (which is the overall GHG benefit/disadvantage); E_{ER} the emissions and resources consumed for energy recovery (modelled with Ecoinvent 3.8: Waste plastic, mixture {CH}| treatment of, municipal incineration); $E_{se,heat}$ and $E_{se,elec}$ are the emissions and resources consumed that would have arisen from specific substituted energy source (as Ecoinvent 3.8: Electricity, medium voltage {NL}| market for Heat, district or industrial, natural gas {RER}| market group for); $X_{ER,heat}$ and $X_{ER,elec}$ is the efficiency of energy recovery (28% for electricity and 9.3% thermal efficiency); LHV is the lower heating value of the material used for energy recovery (Table S.6).

Table S6: different data on LHV of plastics and mixed plastic waste.

Material*	Lower Heating Value (MJ/kg)	Source
**MPW: DKR-350**	32.9<br>*PET: 22.7; PE: 43; PP: 33; PVC: 19*	Calculated based on composition
**MPW**	44	Jeswani et al. (2021)
**Plastics**	23 – 42	Bergsma et al. (2011)

* Mixed-plastic waste contains impurities (0.1 kg/kg MPW).

Crude glycerol

Crude glycerol can be refined to provide the pharmaceutical, food and cosmetic industry with pure glycerol, which is valuable but only economically feasible for large producers[28]. Direct combustion of glycerol is challenging due to its high viscosity and low energy density, therefore, recent developments are made to convert glycerol into fuel, hydrogen, biogas and/or co-generate heat and power[29,30]. The main alternative uses of glycerol are therefore modelled as (Table S.7): purification of glycerol[31] and combustion of biogas, fermented from glycerol[32] to generate electricity and heat[12]. The counterfactual of pure glycerol, i.e. synthetic glycerol, is based on synthetization of propylene via epichlorohydrin and is taken from Ecoinvent (3.8).

Table S7: overview data on alternative uses for crude glycerol.

Heat and power via biogas (fermentation)

Biogas yield	1004.30	m^3/t DM	(Stucki et al., 2011)
per kg crude glycerol	0.80	m^3/kg DM	
CH4 content	50%		(IEA, 2020)
Electricity efficiency	35%		(IEA, 2020)
Thermal efficiency	50%		(IEA, 2020)
MJ per m3 biogas	18	MJ/m^3 for 50% CH4 content	(IEA, 2020)

	Modelled	Amount	unit
Input	Electricity*	0.57	MJ
Input	Heat*	3.47	MJ
Input	Crude glycerol	1	kg
Output	Biogas	0.8034	m3
Input	Biogas	0.8034	m3
Input	Processing (based on: heat and power co-generation, biogas, gas engine)	1	kWh
Input	Processing (based on: heat and power co-generation, biogas, gas engine)	1	MJ
Output	Electricity	5.12	MJ
Output	Heat	7.23	MJ

* Energy consumption per m^3 of biogas: 0.158 kWh of electricity and 3.470 MJ heat per m^3 biogas[32].

**Purification of glycerol**	Glycerol refining: 1.2 kg CO_2 eq./kg crude glycerol[31].
**Synthetic glycerol**	Ecoinvent 3.8: Glycerine {RER}| production, from epichlorohydrin.
**Heat and power (direct combustion)**	LHV glycerol: ~16 MJ/kg. Glycerol has a very high activation energy resulting in an auto-ignition temperature of 370 °C. ***Conclusion****: Direct combustion of glycerol is challenging due to its high viscosity and low energy density.*
**Syngas production (steam reforming)**	Steam reforming producing syngas (white et al., 2018). ***Conclusion****: still in development, not considered as "common use".*

S2 Results

S2.1 Endpoint results

Table S8: ReCiPe 2016 endpoint results for commercial scale (2024) and future scenario (2050) BTX-pathways. BTX = benzene, toluene and xylene.

Product	Endpoint	Unit / kg BTX	Commercial	Future
**MPW-BTX**	Resources	USD (2013)	2.49E-02	1.46E-02
**Bio-BTX**			1.94E-01	1.79E-01
**Fossil-BTX**			5.66E-01	5.65E-01
**MPW-BTX**	Human Health	DALY	4.60E-06	1.39E-06
**Bio-BTX**			5.78E-06	1.82E-06
**Fossil-BTX**			6.15E-06	3.35E-06
**MPW-BTX**	Ecosystems	Species·yr	1.35E-08	4.19E-09
**Bio-BTX**			1.36E-07	1.26E-07
**Fossil-BTX**			1.67E-08	8.40E-09

S2.2 Midpoint results

Table S9: ReCiPe 2016 midpoint results for commercial scale (2024) and future (2050) BTX-pathways. BTX = benzene, toluene and xylene.

Product	Midpoint impact category	Unit / kg BTX	Current	Future
MPW-BTX	Freshwater ecotoxicity	1,4-DBC eq.	1.5E-04	1.1E-04
Bio-BTX			1.9E-03	1.8E-03
Fossil-BTX			1.1E-04	1.0E-04
MPW-BTX	Marine ecotoxicity	1,4-DBC eq.	2.6E-04	2.0E-04
Bio-BTX			4.0E-03	4.0E-03
Fossil-BTX			2.4E-04	2.5E-04
MPW-BTX	Terrestial ecotoxicity	1,4-DBC eq.	6.7E-03	4.0E-03
Bio-BTX			6.9E-01	6.9E-01
Fossil-BTX			2.8E-03	3.4E-03
MPW-BTX	Fossil resource scarcity	kg oil-eq	1.2E-01	4.6E-02
Bio-BTX			6.0E-01	4.9E-01
Fossil-BTX			1.4E+00	1.4E+00
MPW-BTX	Mineral resource scarcity	Kg Cu eq.	9.2E-04	6.2E-04
Bio-BTX			8.7E-03	8.6E-03
Fossil-BTX			3.2E-04	3.4E-04
MPW-BTX	Freshwater eutrophication	kg P eq.	3.5E-05	5.3E-06
Bio-BTX			1.8E-04	1.4E-04
Fossil-BTX			2.5E-05	2.2E-05
MPW-BTX	Marine eutrophication	kg N eq.	5.3E-06	3.7E-06
Bio-BTX			2.5E-04	2.5E-04

Fossil-BTX			3.0E-06	3.0E-06
MPW-BTX	Terrestial acidification	kg SO_2 eq.	1.3E-03	4.5E-04
Bio-BTX			1.1E-02	9.1E-03
Fossil-BTX			4.7E-03	4.6E-03
MPW-BTX	Global warming potential	kg CO_2 eq.	4.6E+00	1.3E+00
Bio-BTX			3.2E+00	-3.7E-01
Fossil-BTX			5.2E+00	2.3E+00
MPW-BTX	Water consumption	m^3	8.0E-03	1.5E-02
Bio-BTX			4.2E-02	6.1E-02
Fossil-BTX			3.2E-02	3.5E-02
MPW-BTX	Ionizing radiation	kBq Co-60 eq.	2.0E-01	3.9E-02
Bio-BTX			1.6E-01	5.5E-02
Fossil-BTX			2.1E-02	1.1E-02
MPW-BTX	Land occupation	m^2·annual crop eq.	8.9E-03	5.7E-03
Bio-BTX			1.4E+01	1.4E+01
Fossil-BTX			6.5E-03	7.6E-03
MPW-BTX	Land transformation	m^2 annual crop eq	2.3E-05	1.5E-05
Bio-BTX			2.2E-04	2.3E-04
Fossil-BTX			6.3E-06	7.4E-06
MPW-BTX	Ozone depletion	kg CFC11 eq.	5.3E-07	4.5E-07
Bio-BTX			3.0E-05	3.0E-05
Fossil-BTX			7.2E-08	6.2E-08
MPW-BTX	Particulate matter formation	kg PM2.5 eq.	4.7E-04	1.6E-04
Bio-BTX			4.4E-03	3.1E-03
Fossil-BTX			1.9E-03	1.8E-03
MPW-BTX	Toxicity, carcinogenic	1,4-DBC eq.	6.2E-03	4.6E-03
Bio-BTX			2.1E-02	1.9E-02
Fossil-BTX			5.8E-03	5.7E-03
MPW-BTX	Toxicity, non-carcinogenic	1,4-DBC eq.	1.6E-02	9.7E-03
Bio-BTX			1.6E-01	1.6E-01
Fossil-BTX			3.5E-03	3.7E-03

Table S10: Table S.9: Environmental Footprint midpoint results commercial scale (2024) BTX-pathways. BTX = benzene, toluene and xylene

**Midpoint impact category**	**Unit**	**Biobased BTX**	**MPW-BTX**	**Fossil-BTX**
Climate change	kg CO2 eq	2.9E+00	4.8E+00	5.2E+00
Ozone depletion	kg CFC11 eq	3.0E-07	3.5E-08	6.1E-10
Ionising radiation	kBq U-235 eq	8.4E-02	1.7E-02	1.2E-04
Photochemical ozone formation	kg NMVOC eq	1.4E-02	1.3E-03	5.1E-03
Particulate matter	disease inc.	8.5E-08	5.5E-09	6.7E-08
Human toxicity, non-cancer	CTUh	8.7E-08	5.9E-09	3.1E-09
Human toxicity, cancer	CTUh	1.3E-09	1.5E-10	4.4E-10
Acidification	mol H+ eq	1.8E-02	1.6E-03	6.2E-03

Eutrophication, freshwater	kg P eq	7.2E-04	3.6E-05	4.7E-07
Eutrophication, marine	kg N eq	1.7E-02	4.5E-04	9.7E-04
Eutrophication, terrestrial	mol N eq	5.6E-02	5.0E-03	1.1E-02
Ecotoxicity, freshwater	CTUe	3.8E+02	8.4E+00	9.7E-01
Land use	Pt	6.9E+02	8.8E-01	4.1E-03
Water use	m3 depriv.	1.5E+00	1.8E-01	1.4E+00
Resource use, fossils	MJ	2.8E+01	8.6E+00	6.1E+01
Resource use, minerals and metals	kg Sb eq	5.3E-06	1.7E-07	2.0E-08

S2.3 Absolute assessments results

Table S11: PB-LCIA results for commercial (2024) and future (2050) scale BTX-pathways. SoSOS = Share of Safe Operating Space.

**Product**	**Planetary Boundary**	**SoSOS current**	**SoSOS future**
**MPW-BTX**	Climate_Change (CO2 concentration)	109	32
**Bio-BTX**	Climate_Change (CO2 concentration)	47	0
**Fossil-BTX**	Climate_Change (CO2 concentration)	115	45
**MPW-BTX**	Climate_Change (Energy imbalance)	103	0.4
**Bio-BTX**	Climate_Change (Energy imbalance)	53	0
**Fossil-BTX**	Climate_Change (Energy imbalance)	109	0.6
**MPW-BTX**	Stratospheric Ozone Depletion	0	0
**Bio-BTX**	Stratospheric Ozone Depletion	0	0
**Fossil-BTX**	Stratospheric Ozone Depletion	0	0
**MPW-BTX**	Ocean Acidification	35	0.1
**Bio-BTX**	Ocean Acidification	15	0
**Fossil-BTX**	Ocean Acidification	37	0.1
**MPW-BTX**	Biochemical flow P	3	0
**Bio-BTX**	Biochemical flow P	5	2
**Fossil-BTX**	Biochemical flow P	2	1
**MPW-BTX**	Biochemical flow N	0	0
**Bio-BTX**	Biochemical flow N	21	21
**Fossil-BTX**	Biochemical flow N	0	0
**MPW-BTX**	Land-system change	0	0
**Bio-BTX**	Land-system change	0	0
**Fossil-BTX**	Land-system change	0	0
**MPW-BTX**	Freshwater use	67	31
**Bio-BTX**	Freshwater use	75	59
**Fossil-BTX**	Freshwater use	9	10
**MPW-BTX**	Biosphere integrity	10	3
**Bio-BTX**	Biosphere integrity	68	61
**Fossil-BTX**	Biosphere integrity	11	5

Table S12: Carrying-capacity based normalization for LCA [33,34]. Total production of BTX per year was taken as 1.22E11 kg (ref).

Impact category	Unit	Normalisation factor	Biobased BTX	MPW-BTX	Fossil BTX
**Ecotoxicity, freshwater**	CTUe	1.3E+14	3.5E-01	7.7E-03	9.0E-04
**Climate change**	kg CO2 eq	6.8E+12	5.2E-02	8.5E-02	9.3E-02
**Eutrophication, freshwater**	kg P eq	5.8E+09	1.5E-02	7.5E-04	9.9E-06
**Resource use, fossils**	MJ	2.2E+14	1.5E-02	4.7E-03	3.3E-02
**Particulate matter**	disease inc.	5.2E+05	1.3E+00	1.3E-03	1.6E-02
**Eutrophication, marine**	kg N eq	2.0E+11	1.0E-02	2.8E-04	5.9E-04
**Land use**	Pt	1.3E+13	6.7E+00	8.4E-03	3.9E-05
**Photochemical ozone formation**	kg NMVOC eq	4.1E+11	4.2E-03	3.9E-04	1.5E-03
**Resource use, minerals and metals**	kg Sb eq	2.2E+08	3.0E-03	9.4E-05	1.1E-05
**Human toxicity, non-cancer**	CTUh	4.1E+06	2.6E-03	1.7E-04	9.2E-05
**Acidification**	mol H+ eq	1.0E+12	2.2E-03	1.9E-04	7.5E-04
**Eutrophication, terrestrial**	mol N eq	6.1E+12	1.1E-03	9.0E-06	1.9E-05
**Water use**	m3 depriv.	1.8E+14	1.0E-03	1.2E-04	9.2E-04
**Human toxicity, cancer**	CTUh	9.6E+05	1.6E-04	1.9E-05	5.6E-05
**Ozone depletion**	kg CFC11 eq	5.4E+08	6.7E-05	7.8E-06	1.4E-07
**Ionising radiation**	kBq U-235 eq	5.3E+14	1.9E-05	4.0E-06	2.9E-08

S2.4 Sensitivity Analysis: electricity market scenarios for 2050

Alongside the baseline scenario for the SSP2 narrative on the electricity market of 2050, two more scenarios were tested: a representative concentration pathway of 1.9 W/m^2 (RCP1.9) as well as 4.5 W/m^2 (RCP4.5) in 2100 [35].

Table S13: ReCiPe 2016 global warming potential results for future (2050) BTX-pathways. The baseline scenario for the SSP2 narrative on the electricity market of 2050: RCP 2.6; a more optimistic scenario of 1.9 W/m2 (RCP1.9); and a more conservative scenario of 4.5 W/m2 (RCP4.5) in 2100. BTX = benzene, toluene and xylene.

Product	Midpoint impact category	Unit / kg BTX	RCP 1.9	RCP 2.6	RCP 4.5
**MPW-BTX**	Global warming potential	kg CO_2 eq.	1.25E+00	1.32E+00	1.51E+00
**Bio-BTX**			-0.46E+00	-0.40E+00	-0.073E+00
**Fossil-BTX**			2.3E+00	2.3E+00	2.4E+00

S2.5 Sensitivity Analysis: plastic recycling scenarios for 2050

Alongside the 2°C-Circulair Economy, we tested less optimistic scenarios of SSP2 baseline and SSP2-RCP 2.6. The SSP2 baseline scenario included 14% chemical or mechanical recycling, 17% landfill stock and 69% littered or incineration with energy recovery. The SSP2-RCP2.6 scenario included 29% recycling, 58% landfill stock and 13% littered or incineration with energy recovery[14].

Table S14: Global warming potential results for future (2050) BTX-pathways applying different recycling scenarios.

Product	Midpoint impact category	Unit / kg BTX	2°C-CE	RCP baseline	RCP 2.6
**MPW-BTX**	Global warming potential	kg CO_2 eq.	1.3	3.2	1.3
**Bio-BTX**			-0.4	1.6	-0.4
**Fossil-BTX**			2.3	4.1	2.3

S2.6 Sensitivity Analysis: Glycerol production from other feedstock

Glycerol production has a big impact on the production of bio-BTX. Figure S.2 shows the environmental impact of different glycerol production routes. These are: glycerol from palm oil (Ecoinvent 3.8: Glycerine {MY} | esterification of palm oil), glycerol from rapeseed oil (Ecoinvent 3.8: Glycerine {Europe without Switzerland} | esterification of rape oil) and glycerol from soybean oil from Brazil (Ecoinvent 3.8: Glycerine {Br} | esterification of soybean oil).

The default of glycerol as a co-product of US soybean bio-diesel has the lowest impact in terms of GHG, acidification, land use and water consumption. Glycerol from rapeseed oil and soybean oil from Brazil have higher GHG emissions, resulting from large impacts from clear-cutting of primary forest to arable land. Likewise, palm oil is known for its large GHG impact from direct land-use changes[36], but also cultivation of the palm fruit bunches themselves is very GHG intensive. For the USA produced glycerol, no LUC emissions were included in line with PAS2050 guidelines[37].

If cultivation requires land use conversion from (tropical) forest to agricultural land, the resulting GHG emissions are high and in regard to the impact of biodiesel and glycerol, it often exceeds fossil-based emission levels [38]. In this regard, and based on the other agricultural relevant impact categories (Figure S.2), glycerol from soybean biodiesel production in the USA has the lowest impact out of the four options. However, the use of other feedstock, e.g. lignin[39], could further decrease bio-BTX's impact, this is discussed in the discussion section.

Moreover, the total GHG emissions of bio-BTX increases from 3 kg CO_2-eq./kg BTX to 4.3 (rape oil), 8.8 (from palm oil) and 11.8 (Brazil) kg CO_2-eq./kg BTX.

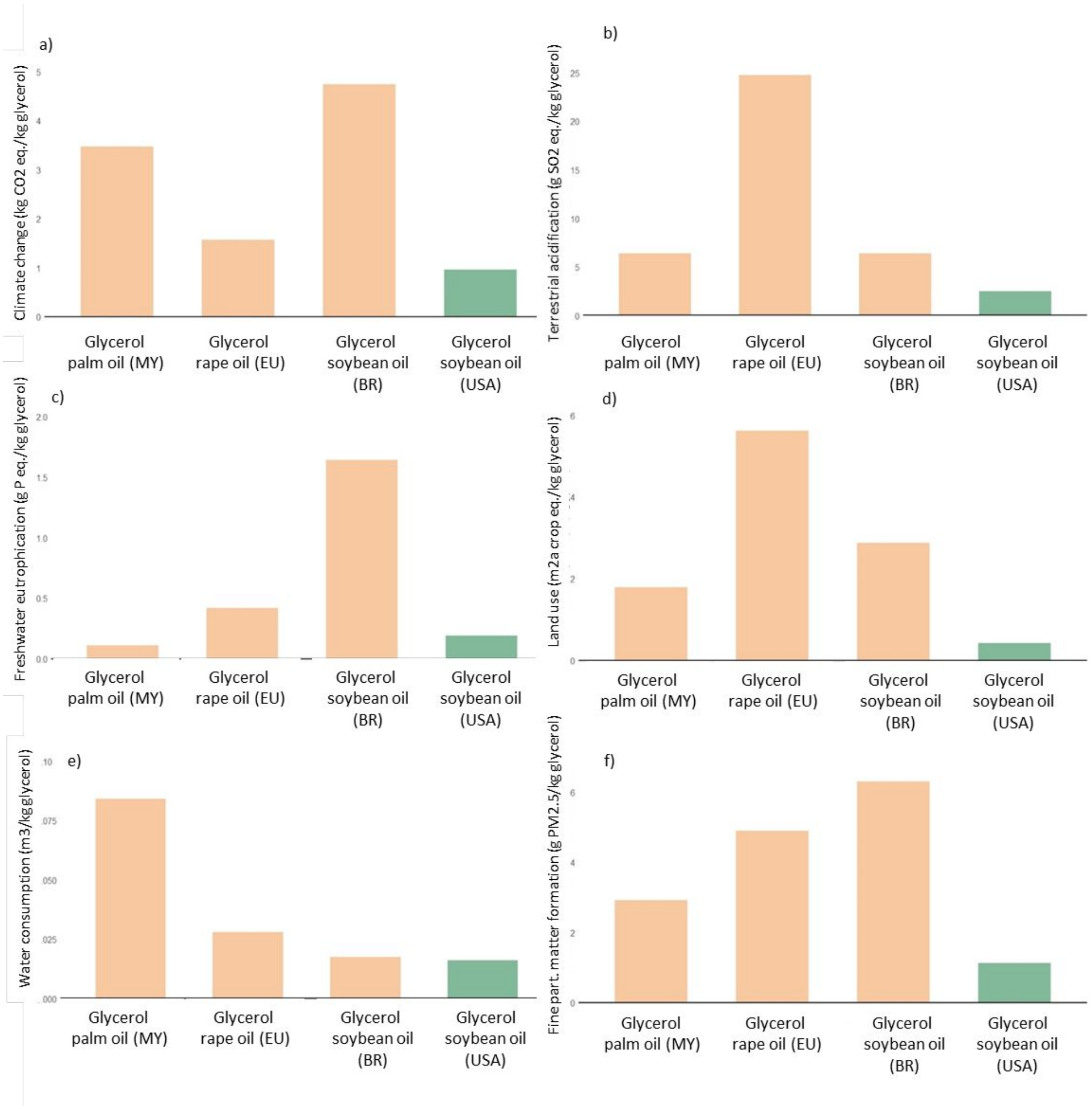

Figure S2: Results of the production of 1 kg of glycerol made from palm oil (left bar), rapeseed oil (one to the right), soybean oil from Brazil (one from the right bar) and soybean oil USA (right bar) on (a) Climate change, (b) Terrestrial acidification, (c) Freshwater eutrophication, (d) Land use, (e) Water consumption, (f) Fine particulate matter formation. Green bar represents the base case glycerol from soybeans (USA).

S2.7 Sensitivity Analysis: Allocation strategies

In the figure down below the results when applying mass, energy and economic allocation on the GHG emissions of the BTX pathways are shown. Other ways to handle multifunctionality were not applied.

Substitution was not applicable because glycerol is a by-product and not the main product, and the co-products in the final step are not marginal - it would lead to skewed impacts. In addition, system expansion was not a solution because the goal of the study is focused on the determination of the impact of the production of 1 kg of BTX[40].

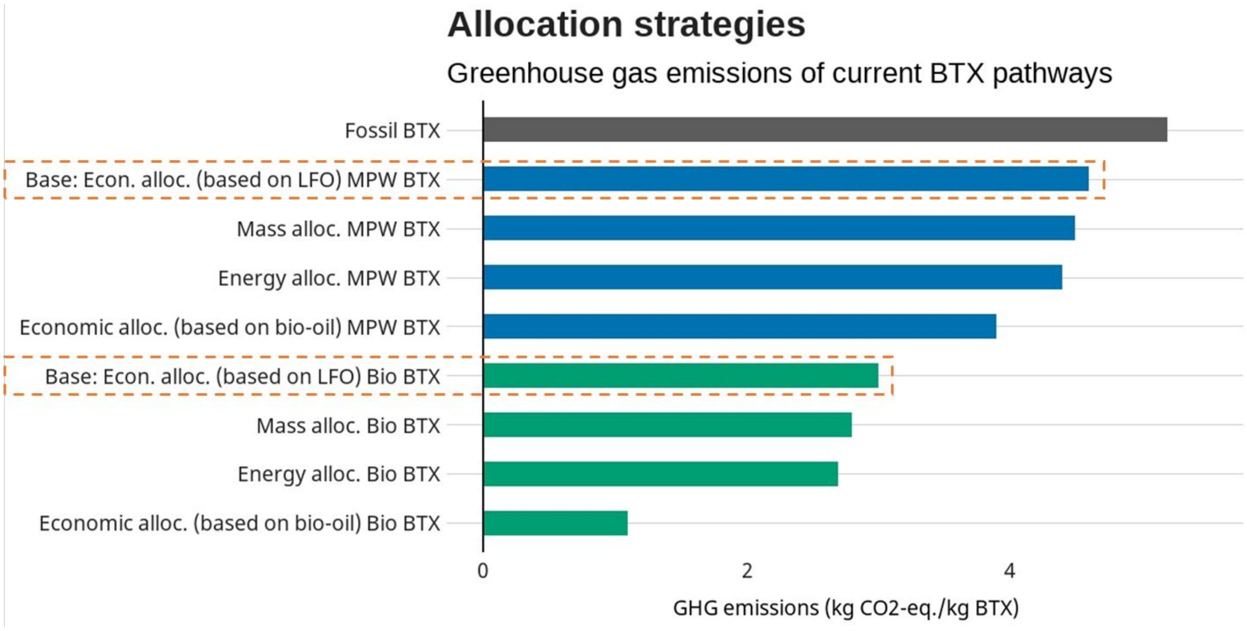

Figure S3: Climate change results of MPW and bio-BTX applying mass, energy and economic allocation. The base scenario is indicated with the orange box. BTX = benzene-toluene-xylene; MPW = Mixed Plastic Waste; LFO = Light Fuel oil.

S3 Discussion

S3.1 Impact other types of biomass feedstock

Table S15: Greenhouse gas (GHG) emissions, freshwater eutrophication, acidification and land use impact of biomass cultivation per kg of BTX, based on yields from literature. The percentage are the in- or decrease in impact compared to soybean cultivation impact. N.A. = not available.

	**Based on a yield of:**	**GHG impact (per kg BTX)**	**Eutrophication**	**Acidification**	**Land use**	***Source***
**Lignin**	10%[i]	N.A	N.A	N.A	N.A	N.A
**Sugarcane bagasse**	12.5%[j]	0.05<br>**-95%**	0.0001<br>**-48%**	0.028<br>**+632%**	2.8<br>**-73%**	*Ecoinvent 3.8*
**Pine wood**	13.3%[k]	0.30<br>**-74%**	0.00006<br>**-71%**	0.00128<br>**-66%**	28.57<br>**+180%**	*Ecoinvent 3.8*
**Sawdust**	6.4%[l]	0.13<br>**-89%**	0.0001<br>**-72%**	0.0006<br>**-85%**	7.03<br>**-31%**	*Ecoinvent 3.8*
**Willow wood**	27%[l]	0.23<br>**-80%**	0.0002<br>**+15%**	0.0015<br>**-60%**	3.52<br>**-66%**	*Ecoinvent 3.8*
**Soybean[n]**	This study	1.17	0.000193	0.00377	10.2	*Ecoinvnt 3.8*

i [41]; j [42]; k [43]; l [44]; m based on mass allocation of 0.25 [45] of sugar from sugarcane production, Brazil. n the impact of soybean cultivation as allocated to glycerol production.

S3.2 Plastic feedstock supply

Table S16: Data and calculations on plastic waste feedstock and it availability for BTX production. BTX = benzene-toluene-xylene.

	**2020**	**2030**	**2050**	**Source**
**Plastic generated annually (Mt)**	512	702	1078	[14]
**Plastic waste generated annually (Mt)**	436	572	953	[14]
**Total plastic waste generated (Mt)**	6300	-	12000	[46]
**BTX production annually (Mt)**	122	143	195	[47,48]
**Plastic waste required to cover annual BTX production**	79%	71%	58%	(yield * BTX production)/annual plastic waste
**Total plastic waste required to cover BTX production from total plastic waste generated**	5.5%	-	4.6%<br>*Up to 2050: 4.84 Gt BTX produced in total => 77% of current generated 6300 Mt waste*	(yield * BTX production)/total plastic waste
**Share of biogenic carbon content**	-	-	If 45%, *an additional 1.5 kg CO_2-eq. per kg MPW-BTX is avoided due to biogenic carbon content (net zero emissions)*	(0.45 • 3.36 kg CO_2)

S3.3 Substitution

To compare to the data of Yang et al.[49], substitution was applied to the bio-BTX product system. This involved substitution of bio-oil, treated as 'light fuel oil {RER}| market for' and the on-site generated electricity as grid electricity. This resulted in credits of in total 2.25 kg CO_2 eq./kg bio-BTX. However, the impact of the process itself increased as well (due to avoidance of allocation), which was mainly affected by an increased impact of glycerol production, i.e., 4.03 kg CO_2 eq./kg bio-BTX.

References

(1) BioBTX. *The future of BTX - BioBTX.* https://biobtx.com/ (accessed 2022-02-03).

(2) Cherubini, F.; Peters, G. P.; Berntsen, T.; Strømman, A. H.; Hertwich, E. CO2 Emissions from Biomass Combustion for Bioenergy: Atmospheric Decay and Contribution to Global Warming. *GCB Bioenergy* **2011**, *3* (5), 413–426. DOI: 10.1111/j.1757-1707.2011.01102.x.

(3) Cherubini, F.; Guest, G.; Strømman, A. H. Application of Probability Distributions to the Modeling of Biogenic CO2 Fluxes in Life Cycle Assessment. *GCB Bioenergy* **2012**, *4* (6), 784–798. DOI: 10.1111/j.1757-1707.2011.01156.x.

(4) Guest, G.; Cherubini, F.; Strømman, A. H. Global Warming Potential of Carbon Dioxide Emissions from Biomass Stored in the Anthroposphere and Used for Bioenergy at End of Life. *J. Ind. Ecol.* **2013**, *17* (1), 20–30. DOI: 10.1111/j.1530-9290.2012.00507.x.

(5) Guest, G.; Bright, R. M.; Cherubini, F.; Strømman, A. H. Consistent Quantification of Climate Impacts Due to Biogenic Carbon Storage across a Range of Bio-Product Systems. *Environ. Impact Assess. Rev.* **2013**, *43*, 21–30. DOI: 10.1016/j.eiar.2013.05.002.

(6) Piccinno, F.; Hischier, R.; Seeger, S.; Som, C. From Laboratory to Industrial Scale: A Scale-up Framework for Chemical Processes in Life Cycle Assessment Studies. *J. Clean. Prod.* **2016**, *135*, 1085–1097. DOI: 10.1016/j.jclepro.2016.06.164.

(7) van der Hulst, M. K.; Huijbregts, M. A. J.; Loon, N.; Theelen, M.; Kootstra, L.; Bergesen, J. D.; Hauck, M. A Systematic Approach to Assess the Environmental Impact of Emerging Technologies: A Case Study for the GHG Footprint of CIGS Solar Photovoltaic Laminate. *J. Ind. Ecol.* **2020**, *24*, 1234–1249. DOI: 10.1111/jiec.13027.

(8) U.S. Department of Energy. *Gas Turbines (DOE CHP Technology Fact Sheet Series)*. Fact Sheet, 2016 | Department of Energy. https://www.energy.gov/eere/amo/downloads/gas-turbines-doe-chp-technology-fact-sheet-series-fact-sheet-2016 (accessed 2022-02-15).

(9) Blok, K. Improving Energy Efficiency by Five Percent and More per Year? *J. Ind. Ecol.* **2004**, *8* (4), 87–99. DOI: 10.1162/1088198043630478.

(10) Bazzanella, A. M.; Ausfelder, F. *Low Carbon Energy and Feedstock for the European Chemical Industry*; 2017. https://dechema.de/en/ Low_carbon_chemical_industry-path-123212,124930.html.

(11) IEA; ICCA; DECHEMA. *Technology Roadmap: Energy and GHG Reductions in the Chemical Industry via Catalytic Processes*; 2013.

(12) Ecoinvent. *Ecoinvent database (Version 3.8)*. https://v37.ecoquery.ecoinvent.org/ (accessed 2021-11-10).

(13) Mendoza Beltran, A.; Cox, B.; Mutel, C.; van Vuuren, D. P.; Font Vivanco, D.; Deetman, S.; Edelenbosch,

O. Y.; Guinée, J.; Tukker, A. When the Background Matters: Using Scenarios from Integrated Assessment Models in Prospective Life Cycle Assessment. *J. Ind. Ecol.* **2020**, *24* (1), 64–79. DOI: 10.1111/jiec.12825.

(14) Stegmann, P.; Daioglou, V.; Londo, M.; van Vuuren, D. P.; Junginger, M. Plastic Futures and Their CO2 Emissions. *Nat. 2022 6127939* **2022**, *612* (7939), 272–276. DOI: 10.1038/s41586-022-05422-5.

(15) PlasticsEurope. *Eco-Profiles and Environmental Product Declarations of the European Plastics Manufacturers: Benzene, Toluene and Xylenes (Aromatics, BTX)*; 2013.

(16) Ryberg, M. W.; Owsianiak, M.; Richardson, K.; Hauschild, M. Z. Development of a Life-Cycle Impact Assessment Methodology Linked to the Planetary Boundaries Framework. *Ecol. Indic.* **2018**, *88*, 250–262. DOI: 10.1016/J.ECOLIND.2017.12.065.

(17) Galán-Martín, Á.; Tulus, V.; Díaz, I.; Pozo, C.; Pérez-Ramírez, J.; Guillén-Gosálbez, G. Sustainability Footprints of a Renewable Carbon Transition for the Petrochemical Sector within Planetary Boundaries. *One Earth* **2021**, *4* (4), 565–583. DOI: 10.1016/J.ONEEAR.2021.04.001.

(18) Tulus, V.; Pérez-Ramírez, J.; Guillén-Gosálbez, G. Planetary Metrics for the Absolute Environmental Sustainability Assessment of Chemicals. *Green Chem.* **2021**, *23* (24), 9881–9893. DOI: 10.1039/D1GC02623B.

(19) Steffen, W.; Richardson, K.; Rockström, J.; Cornell, S. E.; Fetzer, I.; Bennett, E. M.; Biggs, R.; Carpenter, S. R.; De Vries, W.; De Wit, C. A.; Folke, C.; Gerten, D.; Heinke, J.; Mace, G. M.; Persson, L. M.; Ramanathan, V.; Reyers, B.; Sörlin, S. Planetary Boundaries: Guiding Human Development on a Changing Planet. *Science (80-.).* **2015**, *347* (6223). DOI: 10.1126/science.1259855.

(20) Ryberg, M. W.; Owsianiak, M.; Clavreul, J.; Mueller, C.; Sim, S.; King, H.; Hauschild, M. Z. How to Bring Absolute Sustainability into Decision-Making: An Industry Case Study Using a Planetary Boundary-Based Methodology. *Sci. Total Environ.* **2018**, *634*, 1406–1416. DOI: 10.1016/J.SCITOTENV.2018.04.075.

(21) Ryberg, M. W.; Bjerre, T. K.; Nielsen, P. H.; Hauschild, M. Absolute Environmental Sustainability Assessment of a Danish Utility Company Relative to the Planetary Boundaries. *J. Ind. Ecol.* **2021**, *25* (3), 765–777. DOI: 10.1111/JIEC.13075.

(22) Hanafiah, M. M.; Hendriks, A. J.; Huijbregts, M. A. J. Comparing the Ecological Footprint with the Biodiversity Footprint of Products. *J. Clean. Prod.* **2012**, *37*, 107–114. DOI: 10.1016/j.jclepro.2012.06.016.

(23) Wilting, H. C.; Schipper, A. M.; Bakkenes, M.; Meijer, J. R.; Huijbregts, M. A. J. Quantifying Biodiversity Losses Due to Human Consumption: A Global-Scale Footprint Analysis. *Environ. Sci. Technol.* **2017**, *51* (6), 3298–3306. DOI: 10.1021/acs.est.6b05296.

(24) Schipper, A.; Bakkenes, M.; Meijer, J.; Alkemade, R.; Huijbregts, M. The GLOBIO Model. A Technical Description of Version 3.5. **2016**, No. March, 36.

(25) Jeswani, H.; Krüger, C.; Russ, M.; Horlacher, M.; Antony, F.; Hann, S.; Azapagic, A. Life Cycle Environmental Impacts of Chemical Recycling via Pyrolysis of Mixed Plastic Waste in Comparison with Mechanical Recycling and Energy Recovery. *Sci. Total Environ.* **2021**, *769*, 144483. DOI: 10.1016/J.SCITOTENV.2020.144483.

(26) EC. Commission Recommendation of 16.12.2021 on the Use of the Environmental Footprint Methods to Measure and Communicate the Life Cycle Environmental Performance of Products and Organisations. *COM (2021) 9332 final*. Brussels 2021. https://www.consilium.europa.eu/media/40928/st12791-en19.pdf (accessed 2022-06-06).

(27) Corsten, M.; Worrell, E.; Rouw, M.; Van Duin, A. The Potential Contribution of Sustainable Waste Management to Energy Use and Greenhouse Gas Emission Reduction in the Netherlands. *Resour. Conserv. Recycl.* **2013**, *77*, 13–21. DOI: 10.1016/J.RESCONREC.2013.04.002.

(28) Kaur, J.; Sarma, A. K.; Jha, M. K.; Gera, P. Valorisation of Crude Glycerol to Value-Added Products: Perspectives of Process Technology, Economics and Environmental Issues. *Biotechnol. Reports* **2020**, *27*,

e00487. DOI: 10.1016/J.BTRE.2020.E00487.

(29) Tan, H. W.; Abdul Aziz, A. R.; Aroua, M. K. Glycerol Production and Its Applications as a Raw Material: A Review. *Renew. Sustain. Energy Rev.* **2013**, *27*, 118–127. DOI: 10.1016/J.RSER.2013.06.035.

(30) Monteiro, M. R.; Kugelmeier, C. L.; Pinheiro, R. S.; Batalha, M. O.; da Silva César, A. Glycerol from Biodiesel Production: Technological Paths for Sustainability. *Renew. Sustain. Energy Rev.* **2018**, *88*, 109–122. DOI: 10.1016/J.RSER.2018.02.019.

(31) Cespi, D.; Passarini, F.; Mastragostino, G.; Vassura, I.; Larocca, S.; Iaconi, A.; Chieregato, A.; Dubois, J. L.; Cavani, F. Glycerol as Feedstock in the Synthesis of Chemicals: A Life Cycle Analysis for Acrolein Production. *Green Chem.* **2014**, *17* (1), 343–355. DOI: 10.1039/C4GC01497A.

(32) Stucki, M.; Jungbluth, N.; Leuenberger, M. *Life Cycle Assessment of Biogas Production from Different Substrates*; 2011.

(33) Sala, S.; Crenna, E.; Secchi, M.; Sanyé-Mengual, E. Environmental Sustainability of European Production and Consumption Assessed against Planetary Boundaries. *J. Environ. Manage.* **2020**, *269* (April). DOI: 10.1016/j.jenvman.2020.110686.

(34) Bjørn, A.; Hauschild, M. Z. Introducing Carrying Capacity-Based Normalisation in LCA: Framework and Development of References at Midpoint Level. *Int. J. Life Cycle Assess.* **2015**, *20* (7), 1005–1018. DOI: 10.1007/S11367-015-0899-2/TABLES/1.

(35) Stehfest, E.; van Vuuren, D.; Kram, T.; Bouwman, L.; Alkemade, R.; Bakkenes, M.; Biemans, H.; Bouwman, A.; den Elzen, M.; Janse, J.; Lucas, P.; van Minnen, J.; Müller, C.; Gerdien Prins, A. *Integrated Assessment of Global Environmental Change with IMAGE 3.0 - Model Description and Policy Applications*; The Hague, 2014. https://www.pbl.nl/en/publications/integrated-assessment-of-global-environmental-change-with-IMAGE-3.0 (accessed 2022-03-14).

(36) Achten, W. M. J.; Verchot, L. V. Implications of Biodiesel-Induced Land-Use Changes for CO2 Emissions: Case Studies in Tropical America, Africa, and Southeast Asia. *Ecol. Soc.* **2011**, *16* (4). DOI: 10.5751/ES-04403-160414.

(37) PAS2050. *PAS 2050: 2011 - Specification for the assessment of the life cycle greenhouse gas emissions of goods and services*. DOI: DOI: 978.0.580.71382.8.

(38) Uusitalo, V.; Väisänen, S.; Havukainen, J.; Havukainen, M.; Soukka, R.; Luoranen, M. Carbon Footprint of Renewable Diesel from Palm Oil, Jatropha Oil and Rapeseed Oil. *Renew. Energy* **2014**, *69*, 103–113. DOI: 10.1016/j.renene.2014.03.020.

(39) Fan, L.; Ruan, R.; Li, J.; Ma, L.; Wang, C.; Zhou, W. Aromatics Production from Fast Co-Pyrolysis of Lignin and Waste Cooking Oil Catalyzed by HZSM-5 Zeolite. *Appl. Energy* **2020**, *263*, 114629. DOI: 10.1016/J.APENERGY.2020.114629.

(40) Moretti, C.; Corona, B.; Edwards, R.; Junginger, M.; Moro, A.; Rocco, M.; Shen, L. Reviewing ISO Compliant Multifunctionality Practices in Environmental Life Cycle Modeling. *Energies 2020, Vol. 13, Page 3579* **2020**, *13* (14), 3579. DOI: 10.3390/EN13143579.

(41) Vural Gursel, I.; Dijkstra, J. W.; Huijgen, W. J. J.; Ramirez, A. Techno-Economic Comparative Assessment of Novel Lignin Depolymerization Routes to Bio-Based Aromatics. *Biofuels, Bioprod. Biorefining* **2019**, *13* (4), 1068–1084. DOI: 10.1002/bbb.1999.

(42) Ghorbannezhad, P.; Firouzabadi, M. D.; Ghasemian, A.; de Wild, P. J.; Heeres, H. J. Sugarcane Bagasse Ex-Situ Catalytic Fast Pyrolysis for the Production of Benzene, Toluene and Xylenes (BTX). *J. Anal. Appl. Pyrolysis* **2018**, *131*, 1–8. DOI: 10.1016/j.jaap.2018.02.019.

(43) Mendes, F. L.; Ximenes, V. L.; de Almeida, M. B. B.; Azevedo, D. A.; Tessarolo, N. S.; de Rezende Pinho, A. Catalytic Pyrolysis of Sugarcane Bagasse and Pinewood in a Pilot Scale Unit. *J. Anal. Appl. Pyrolysis* **2016**, *122*, 395–404. DOI: 10.1016/J.JAAP.2016.08.001.

(44) Balasundram, V.; Ibrahim, N.; Kasmani, R. M.; Isha, R.; Hamid, M. K. A.; Hasbullah, H. Catalytic Upgrading of Biomass-Derived Pyrolysis Vapour over Metal-Modified HZSM-5 into BTX: A Comprehensive Review. *Biomass Conversion and Biorefinery*. Springer August 5, 2020, pp 1–28. DOI: 10.1007/s13399-020-00909-5.

(45) Carpio, L. G. T.; Simone de Souza, F. Optimal Allocation of Sugarcane Bagasse for Producing Bioelectricity and Second Generation Ethanol in Brazil: Scenarios of Cost Reductions. *Renew. Energy* **2017**, *111*, 771–780. DOI: 10.1016/J.RENENE.2017.05.015.

(46) Geyer, R.; Jambeck, J. R.; Law, K. L. Production, Use, and Fate of All Plastics Ever Made. *Sci. Adv.* **2017**, *3* (7). DOI: 10.1126/SCIADV.1700782/SUPPL_FILE/1700782_SM.PDF.

(47) IEA. *The Future of Petrochemicals: Towards More Sustainable Plastics and Fertilisers*; 2018. DOI: 10.1787/9789264307414-en.

(48) IEA. *Chemicals* . Paris https://www.iea.org/reports/chemicals. https://www.iea.org/reports/chemicals (accessed 2020-10-19).

(49) Yang, F.; Meerman, H.; Zhang, Z.; Jiang, J.; Faaij, A. Integral Techno-Economic Comparison and Greenhouse Gas Balances of Different Production Routes of Aromatics from Biomass with CO2 Capture. *J. Clean. Prod.* **2022**, *372*, 133727. DOI: 10.1016/J.JCLEPRO.2022.133727.
